# Synthesis and Antimicrobial Activity of Some Novel 5-Alkyl-6-Substituted Uracils and Related Derivatives

**DOI:** 10.3390/molecules16064764

**Published:** 2011-06-08

**Authors:** Abdulghafoor A. Al-Turkistani, Omar A. Al-Deeb, Nasser R. El-Brollosy, Ali A. El-Emam

**Affiliations:** Department of Pharmaceutical Chemistry, College of Pharmacy, King Saud University, Riyadh 11451, Saudi Arabia

**Keywords:** synthesis, 5-alkyluracils, 8-alkyltetrazolo[1,5-*f*]pyrimidine-5,7(3*H*,6*H*)-dione, antimicrobial activity

## Abstract

6-Chloro-5-ethyl-, *n*-propyl- and isopropyluracils **5a**-**c** were efficiently prepared from the corresponding 5-alkybarbituric acids **3a**-**c***via* treatment with phosphorus oxychloride and *N*,*N*-dimethylaniline to yield the corresponding 5-alkyl-2,4,6-trichloro-pyrimidines **4a**-**c**, which were selectively hydrolyzed by heating in 10% aqueous sodium hydroxide for 30 minutes. The reaction of compounds **5a**-**c** with 1-substituted piperazines yielded the corresponding 5-alkyl-6-(4-substituted-1-piperazinyl)uracils **6a**-**j**. The target 8-alkyltetrazolo[1,5-*f*]pyrimidine-5,7(3*H*,6*H*)-diones **7a**-**c** were prepared *via* the reaction of **5a**-**c** with sodium azide. Compounds **6a**-**j** and **7a**-**c** were tested for *in vitro* activities against a panel of Gram-positive and Gram-negative bacteria and the yeast-like pathogenic fungus *Candida albicans*. Compound **6h** displayed potent broad-spectrum antibacterial activity, while compound **6b** showed moderate activity against the Gram-positive bacteria. All the tested compounds were practically inactive against *Candida albicans*.

## 1. Introduction

The development of new chemotherapeutic agents is becoming the major interest in many academic and industrial research laboratories all over the world with the aim to discover newer, more potent molecules, with higher specificity and reduced toxicity than the existing ones. In addition, the various types of resistant microorganisms that are being discovered nowadays are becoming a great challenge for scientists. Uracils occupy a distinct and unique place in medicine. The chemotherapeutic efficacy of uracil and pyrimidine derivatives is related to their ability to inhibit vital enzymes responsible for DNA biosynthesis such as dihydrofolate reductase (DHFR), thymidylate synthetase (TSase), thymidine phosphorylase (TPase) and reverse transcriptase (RTase). A large array of uracil non-nucleoside derivatives possess a variety chemotherapeutic properties. These properties include anticancer [1,2,3,4,5], antiviral [6,7,8,9,10,11,12,13,14] and antimicrobial activities [15,16,17,18,19]. Moreover, several bis(heteroaryl)piperazine derivatives (BHAP) were introduced as potent anti-HIV drugs [20,21]. In addition, fused pyrimidines were reported to exhibit important biological activities [22,23,24].

In continuation to our interest in the chemical and pharmacological properties of uracil derivatives [10,11,12,13,14,25,26,27,14,25], we report herein the synthesis of a new series of 6-(4-substituted-1-piperazinyl)-5-alkyluracils and 8-alkyltetrazolo[1,5-*f*]pyrimidine-5,7(3*H*,6*H*)-diones as potential antimicrobial agents.

## 2. Results and Discussion

### 2.1. Chemistry

**Scheme 1:**
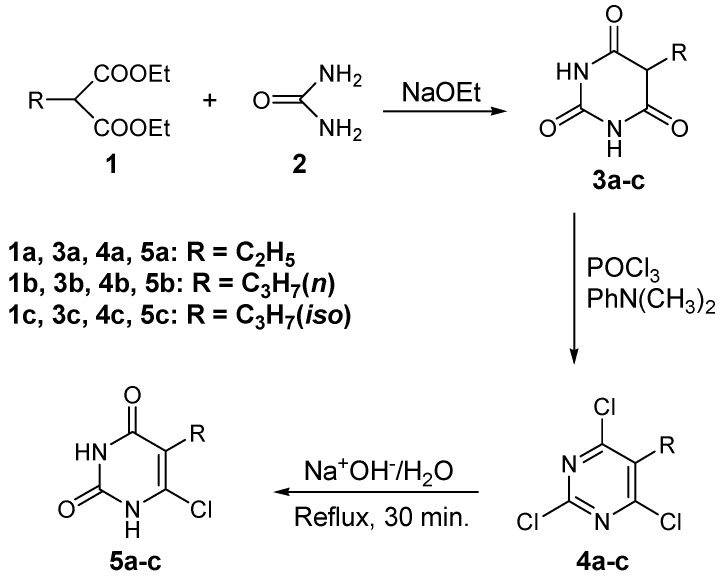
Synthesis of compounds **5a**-**c**.

The conversion of barbituric acid and its 5-substituted derivatives to the corresponding 6-chlorouracils has been reported by several authors. Kaul *et al.* [28] and Koroniak *et al.* [29] reported the preparation of 5-alkyl-6-chlorouracils *via* the one-pot reaction of the corresponding 5-alkyl-barbituric acid with phospophorus oxychloride and trace amounts of water. This one-pot reaction yields considerable amounts of 5-alkyl-2,4,6-trichloropyrimines as by-products. In the present investigation, 5-(ethyl, *n*-propyl or isopropyl)-barbituric acids **3a**-**c** were prepared in good yields by the condensation of the appropriate diethyl alkylmalonate **1a**-**c** with urea (**2**) in dry methanol, in the presence of sodium methoxide [29]. The 5-alkylbarbituric acids **3a**-**c** were further reacted with POCl_3_ and *N*,*N*-dimethylaniline to yield the corresponding 5-alkyl-2,4,6-trichloropyrimidines **4a**-**c**, which were selectively hydrolyzed by heating in aqueous sodium hydroxide for 30 minutes to yield the target derivatives **5a**-**c** in good overall yields (Scheme 1).

**Scheme 2:**
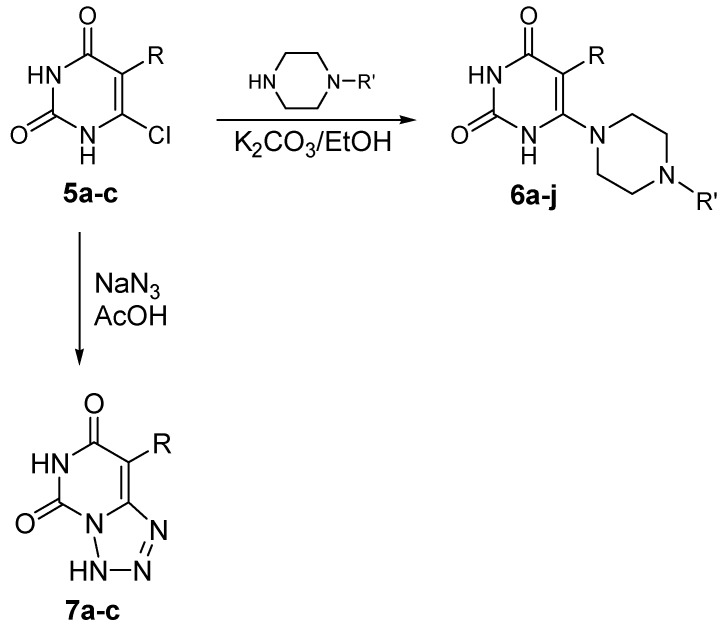
Synthesis of compounds **6a**-**j** and **7a**-**c**.

The reaction of compounds **5a**-**c** with some 1-substituted piperazines in ethanol in the presence of potassium carbonate yielded the target 5-alkyl-6-(4-substituted-1-piperazinyl)uracils **6a**-**j** in 51-91% yields. Compounds **5a**-**c** were reacted with aqueous sodium azide solution in acetic acid *via* heating for 4 hours to yield the target 8-alkyltetrazolo[1,5-*f*]pyrimidine-5,7(3*H*,6*H*)-diones **7a**-**c**. The reaction is thought to proceed through nucleophilic substitution of the chlorine atom to yield the 6-azido derivatives, which subsequently cyclized to give compounds **7a**-**c** (Scheme 2, Table 1). The structures of the newly synthesized compounds were confirmed on the basis of their elemental analysis and IR, ^1^H-NMR, ^13^C-NMR and mass spectral data.

**Table 1:** Melting points, yield percentages, molecular formulae and molecular weights of newly synthesized compounds **6a**-**j** and **7a**-**c**.

Comp. No.	R	R’	Mp (°C)	Yield (%)	Molecular Formula (Mol. Wt.)
**6a**	C_2_H_5_	COOC_2_H_5_	134-6	64	C_13_H_20_N_4_O_4_ (296.32)
**6b**	C_2_H_5_	C_6_H_5_	173-5	51	C_16_H_20_N_4_O_2_ (300.36)
**6c**	C_2_H_5_	2-CH_3_OC_6_H_4_	166-8	65	C_17_H_22_N_4_O_3_ (330.38)
**6d**	C_3_H_7_(*n*)	C_2_H_5_	148-50	62	C_13_H_22_N_4_O_2_ (266.34)
**6e**	C_3_H_7_(*n*)	COOC_2_H_5_	161-3	70	C_14_H_22_N_4_O_4_ (310.35)
**6f**	C_3_H_7_(*n*)	C_6_H_5_	176-8	88	C_17_H_22_N_4_O_2_ (314.38)
**6g**	C_3_H_7_(*n*)	2-CH_3_OC_6_H_4_	169-71	85	C_18_H_24_N_4_O_3_ (344.41)
**6h**	C_3_H_7_(*n*)	3-CF_3_C_6_H_4_	174-6	76	C_18_H_21_F_3_N_4_O_2_ (382.38)
**6i**	C_3_H_7_(*iso*)	C_6_H_5_	192-4	90	C_17_H_22_N_4_O_2_ (314.38)
**6j**	C_3_H_7_(*iso*)	2-CH_3_OC_6_H_4_	188-90	91	C_18_H_24_N_4_O_3_ (344.41)
**7a**	C_2_H_5_	-	215-7 (dec.)	52	C_6_H_7_N_5_O_2_ (181.06)
**7b**	C_3_H_7_(*n*)	-	252-4 (dec.)	59	C_7_H_9_N_5_O_2_ (195.18)
**7c**	C_3_H_7_(*iso*)	-	242-4 (dec.)	55	C_7_H_9_N_5_O_2_ (195.18)

### 2.2. Antimicrobial Testing

The newly synthesized compounds **6a**-**j** and **7a**-**c** were tested for their *in vitro* growth inhibitory activity against the standard strains of the Institute of Fermentation of Osaka (IFO) namely; *Staphylococcus aureus* IFO 3060, *Bacillus subtilis* IFO 3007, *Micrococcus luteus* IFO 3232 (Gram-positive bacteria), *Escherichia coli* IFO 3301, *Pseudomonas aeuroginosa* IFO 3448 (Gram-negative bacteria), and the yeast-like pathogenic fungus *Candida albicans* IFO 0583. The primary screening was carried out using the agar disc-diffusion method using Müller-Hinton agar medium [30]. The results of the preliminary antimicrobial testing of compounds **6a**-**j** and **7a**-**c** (200 μg/disc), the antibacterial antibiotic ampicillin trihydrate (100 μg/disc) and the antifungal drug clotrimazole (100 μg/disc) are shown in Table 2.

**Table 2:** Antimicrobial activity of compounds **6a**-**j** and **7a**-**c **(200 μg/8 mm disc), the broad spectrum antibacterial antibiotics gentamicin (100 μg/8 mm disc), ampicillin (100 μg/8 mm disc) and the antifungal drug clotrimazole (100 μg/8 mm disc) against *Staphylococcus aureus* IFO 3060 (*SA*), *Bacillus subtilis* IFO 3007 (*BS*), *Micrococcus luteus* IFO 3232 (*ML*), *Escherichia coli* IFO 3301(*EC*), *Pseudomonas aeuroginosa* IFO 3448 (*PA*), and *Candida albicans* IFO 0583 (*CA*).

Comp. No.	Diameter of Growth Inhibition Zone (mm) *					
	*SA*	*BS*	*ML*	*EC*	*PA*	*C A*
**6a**	-	-	-	-	-	-
**6b**	12	14	15	-	-	-
**6c**	-	-	13	-	-	-
**6d**	-	-	15	-	-	-
**6e**	-	-	11	-	-	-
**6f**	-	-	11	-	-	-
**6g**			13			-
**6h**	22(4) **	26(2) **	28(1) **	20(4) **	16(16) **	-
**6i**	-	-	12	-	-	-
**6j**	-	-	14	-	-	-
**7a**	-	-	-	-	-	-
**7b**	-	-	-	-	-	-
**7c**	-	-	-	-	-	-
**Gentamicin**	26(2) **	25(2) **	18(2) **	20(0.5) **	19(1) **	NT
**Ampicillin**	23(2) **	21(0.5) **	19(2) **	17(2) **	16(2) **	NT
**Clotrimazole**	NT	NT	NT	NT	NT	21

* (-): Inactive (inhibition zone < 10 mm). (NT): Not tested. ** The figures shown in parentheses represent the MIC values (μg/mL).

The results revealed that only compound **6h** showed marked broad-spectrum inhibitory activity against the tested bacteria, while compound **6b** displayed limited activity against the tested Gram-positive bacteria. In addition, all the tested compounds were inactive against the yeast-like pathogenic fungus *Candida albicans*. According to the results of the antimicrobial activity, it seems difficult to abstract definite structure-activity relationship. However, we can conclude that the Gram-positive bacteria *Micrococcus luteus* are generally sensitive to the 6-piperazino uracils derivatives **6a**-**j**. In general, the antibacterial activity seemed to be dependent on the nature of 6-subtituents rather the basic skeleton of the molecules. Within the 6-piperazino series **6a**-**j**, the highest activity was observed with the 3-trifluoromethylphenyl-1-piperazinyl derivative **6h** which endows potent broad-spectrum antibacterial activity. In addition, it would be concluded that the synthesized compounds are not suitable candidate for antifungal activity. The minimal inhibitory concentration (MIC) for the active compound **6h** against the same microorganism used in the primary screening was carried out using the microdilution susceptibility method in Müller-Hinton Broth and Sabouraud Liquid Medium [31]. The MIC of compound **6h**, the antibacterial antibiotics ampicillin trihydrate and gentamicin which are shown in Table 2, were in accordance with the results obtained in the primary screening.

## 3. Experimental

### 3.1. General

Melting points (°C) were measured in open glass capillaries using a Branstead 9001 electrothermal melting point apparatus and are uncorrected. NMR spectra were obtained on a Bruker AC 500 Ultra Shield NMR spectrometer (Fällanden, Switzerland) operating at 500.13 MHz for ^1^H and 125.76 MHz for ^13^C; the chemical shifts are expressed in δ (ppm) downfield from tetramethylsilane (TMS) as internal standard; coupling constants (*J*) are expressed in Hz. Electrospray ionization mass spectra (ESI-MS) were recorded on an Agilent 6410 Triple Quad tandem mass spectrometer at 4.0 and 3.5 kV for positive and negative ions, respectively. Elemental analyses (C, H, N, S) were in full agreement with the proposed structures within ±0.4% of the theoretical values. Monitoring the reactions and checking the purity of the final products were carried out by thin layer chromatography (TLC) using silica gel precoated aluminum sheets (60 F_254_, Merck) and visualization with ultraviolet light (UV) at 365 and 254 nm. The bacterial strains and *Candida albicans* fungus were obtained from the Institute of Fermentation of Osaka (IFO), Osaka, Japan. The reference drugs ampicillin trihydrate (CAS 7177-48-2) and clotrimazole (CAS 23593-75-1) were obtained from Sigma-Aldrich Chemie GmbH (Taufkirchen, Germany).

### 3.2. 5-Alkylbarbituric Acids **3a-c** [29]

Methanolic sodium methoxide solution was prepared by portionwise addition of sodium metal (10.35 gm, 0.45 gm-atom) to dry methanol (200 mL) with continuous stirring over a period of 30 minutes. The appropriate diethyl alkylmalonate **1a**-**c** (0.3 mol) was then added dropwise at room temperature and the mixture was heated under reflux for 30 minutes and cooled to room temperature. Urea (**2**, 18 gm, 0.3 mole) was added at once and the reaction mixture was heated under reflux for 10 hours. The mixture was distilled *in vacuo* and the solid residue was dissolved in water (250 mL). The solution was then treated with concentrated hydrochloric acid to pH 2-3 and refrigerated overnight. The precipitated product was filtered, washed with cold water (100 mL) and dried at 80 °C. The products **3a**-**c** which were pure enough (TLC CH_3_Cl:MeOH 8:2 v/v) were used in the second reaction without further purification. Compound **3a**: M.p.: 201-2 °C, Yield: 82%. Compound **3b**: M.p.: 210-1 °C, Yield: 95%. Compound **3c**: M.p.: 216-8 °C, Yield: 76%.

### 3.3. 5-Alkyl-6-Chlorouracils **5a-c**

The appropriate 5-alkybarbituric acid **3a**-**c** (0.05 mol) was added portionwise to a mixture of phosphorus oxychloride (19.2 mL) and *N*,*N*-dimethylaniline (10.3 mL) over a period of 10 minutes with stirring. The mixture was then heated under reflux for one hour. On cooling, the mixture was poured onto crushed ice (200 gm), stirred for 30 minutes and extracted with diethyl ether (2 × 200 mL). The ethereal extract was dried over anhydrous sodium sulphate and evaporated under vacuum at room temperature to yield the crude 5-alkyl-2,4,6-trichloropyrimidines **4a**-**c** as white waxy solids. 10% sodium hydroxide (20 mL) was then added to the crude product and the mixture was heated under reflux for 30 minutes. On cooling, the mixture was acidified with hydrochloric acid to pH 1-2 and the separated precipitate was filtered, washed with cold water and crystallized from ethanol.

*6-Chloro-5-ethyluracil* (**5a**): M.p.: 228-10 °C, Yield: 7.25 gm (83%). ^1^H-NMR (DMSO-d_6_): δ 0.95 (t, 3H, CH_2_C***H***_3_, *J* = 7.5 Hz), 2.86 (q, 2H, C***H***_2_CH_3_, *J* = 7.5 Hz), 11.32 (s, 1H, NH), 11.82 (s, 1H, NH). ^13^C-NMR: 17.06 (CH_2_***C***H_3_), 19.08 (***C***H_2_CH_3_), 111.86 (C-5), 141.08 (C-6), 150.10 (C=O), 163.20 (C=O). ESI-MS, *m/z* (Rel. Int.): 173.1 (M^−^, 100), 175.1 (M^-^ + 2, 34).

*6-Chloro-5-(n-propyl)uracil* (**5b**): M.p.: 238-9 °C, Yield: 8.12 gm (86%). ^1^H-NMR (DMSO-d_6_): δ 0.85 (t, 3H, CH_2_C***H***_3_, *J* = 7.5 Hz), 1.37-1.44 (m, 2H, C***H***_2_CH_3_), 2.26 (t, 2H, C***H***_2_CH_2_, *J* = 7.5 Hz), 11.31 (s, 1H, NH), 11.83 (s, 1H, NH). ^13^C-NMR: 14.05 (CH_2_***C***H_3_), 21.45 (***C***H_2_CH_3_), 27.45 (***C***H_2_CH_2_CH_3_), 110.49 (C-5), 141.49 (C-6), 150.10 (C=O), 163.35 (C=O). ESI-MS, *m/z* (Rel. Int.): 187.1 (M^−^, 100), 189.1 (M^−^ + 2, 31).

*6-Chloro-5-(iso-propyl)uracil* (**5c**): M.p.: 257-9 °C, Yield: 6.98 gm (74%). ^1^H-NMR (DMSO-d_6_): δ 1.14 (d, 6H, CH_3_, *J* = 7.2 Hz), 2.51-2.63 (m, 1H, CH), 11.22 (s, 1H, NH), 11.79 (s, 1H, NH). ^13^C-NMR: 20.02 (CH_3_), 26.52 (CH), 113.95 (C-5), 140.95 (C-6), 149.75 (C=O), 162.75 (C=O). ESI-MS, *m/z* (Rel. Int.): 187.1 (M^−^, 100), 189.1 (M^−^ + 2, 35).

### 3.4. 5-Alkyl-6-(4-Substituted-1-Piperazinyl)uracils **6a-j**

A mixture of the appropriate 6-chlorouracil **5a**-**c** (0.002 mol), the appropriate 1-substituted piperazine (0.002 mol) and anhydrous potassium carbonate (0.28 gm, 0.002 mol), in ethanol (8 mL), was heated under reflux for 6 hours. After cooling the solvent was then distilled off *in vacuo*, and water (10 mL) was added to the residue. The separated precipitate was filtered, washed with cold water, dried and crystallized from ethanol.

*6-(4-Ethoxycarbonyl-1-piperazinyl)-5-ethyluracil* (**6a**): ^1^H-NMR (DMSO-d_6_): δ 0.93 (t, 3H, CH_2_C***H***_3_, *J* = 7.0 Hz), 1.17 (t, 3H, OCH_2_C***H***_3_, *J* = 7.0 Hz), 2.27 (q, 2H, C***H***_2_CH_3_, *J* = 7.0 Hz), 2.90 (s, 4H, piperazine-H), 3.45 (s, 4H, piperazine-H), 4.03 (q, 2H, OC***H***_2_CH_3_, *J* = 7.0 Hz), 8.05 (br. s, 2H, NH). ^13^C-NMR: 13.05 (CH_2_***C***H_3_), 14.46 (***C***H_2_CH_3_), 18.89 (OCH_2_***C***H_3_), 42.22, 43.63 (piperazine-C), 60.87 (O***C***H_2_CH_3_), 108.46 (C-5), 149.21, 153.41, 163.92 (C-2, C-6 & C-4), 154.49 (ester C=O). ESI-MS, *m/z* (Rel. Int.): 295.2 (M^−^, 100).

*6-(4-Phenyl-1-piperazinyl)-5-ethyluracil* (**6b**): ^1^H-NMR (DMSO-d_6_): δ 0.92 (t, 3H, CH_3_, *J* = 6.8 Hz), 2.28 (d, 2H, CH_2_, *J* = 6.8 Hz), 3.14 (s, 4H, piperazine-H), 3.26 (s, 4H, piperazine-H), 6.81-6.96 (m, 3H, Ar-H), 7.22 (d, 2H, Ar-H, *J* = 6.5 Hz), 8.66 (br. s, 2H, NH). ^13^C-NMR: 13.75 (CH_3_), 19.52 (CH_2_), 43.98, 47.27 (piperazine-C), 107.96 (C-5), 116.26, 119.96, 129.46, 151.06 (Ar-C), 152.77, 155.29, 164.87 (C-2, C-6 & C-4). ESI-MS, *m/z* (Rel. Int.): 299.2 (M^−^, 100).

*6-[4-(2-Methoxyphenyl)-1-piperazinyl)]-5-ethyluracil* (**6c**): ^1^H-NMR (DMSO-d_6_): δ 0.90 (t, 3H, CH_2_C***H***_3_, *J* = 7.0 Hz), 2.26 (q, 2H, CH_2_, *J* = 7.0 Hz), 3.42-3.46 (m, 8H, piperazine-H), 3.79 (OCH_3_), 9.82 (s, 2H, NH). ^13^C-NMR: 13.80 (CH_3_), 19.27 (CH_2_), 44.25, 46.51 (piperazine-C), 55.98 (OCH_3_), 104.33 (C-5), 156.77, 159.13, 165.53 (C-2, C-6 & C-4). ESI-MS, *m/z* (Rel. Int.): 331.2 (M^+^, 100).

*6-(4-Ethyl-1-piperazinyl)-5-(n-propyl)uracil* (**6d**): ^1^H-NMR (CDCl_3_): δ 0.74 (t, 3H, CH_2_CH_2_C***H***_3_, *J* = 7.0 Hz), 0.98 (t, 3H, CH_2_C***H***_3_, *J* = 7.0 Hz), 1.29-1.33 (m, 2H, CH_2_C***H***_2_CH_3_), 2.23 (t, 2H, C***H***_2_CH_2_CH_3_, *J* = 7.0 Hz), 2.35 (q, 2H, C***H***_2_CH_3_, *J* = 7.0 Hz), 2.73 (br. s, 4H, piperazine-H), 3.06 (br. s, 4H, piperazine-H), 8.09 (br. s, 2H, NH). ^13^C-NMR: 9.67 (CH_2_CH_2_***C***H_3_), 12.91 (CH_2_***C***H_3_), 21.23 (CH_2_***C***H_2_CH_3_), 27.30 (***C***H_2_CH_2_CH_3_), 42.71, 49.34 (piperazine-C), 51.65 (***C***H_2_CH_3_), 108.77 (C-5), 157.56, 159.21, 167.39 (C-2, C-6 & C-4). ESI-MS, *m/z* (Rel. Int.): 267.2 (M^+^, 100).

*6-(4-Ethoxycarbonyl-1-piperazinyl)-5-(n-propyl)uracil* (**6e**): ^1^H-NMR (DMSO-d_6_): δ 0.95 (t, 3H, CH_2_CH_2_C***H***_3_, *J* = 7.0 Hz), 1.32-1.34 (m, 2H, CH_2_C***H***_2_CH_3_), 1.72 (t, 3H, OCH_2_C***H***_3_, *J* = 7.0 Hz), 2.25 (q, 2H, C***H***_2_CH_2_CH_3_, *J* = 7.0 Hz), 2.92 (s, 4H, piperazine-H), 3.46 (s, 4H, piperazine-H), 4.03 (q, 2H, OC***H***_2_CH_3_, *J* = 7.0 Hz), 8.13 (br. s, 2H, NH). ^13^C-NMR: 13.64 (CH_2_CH_2_***C***H_3_), 14.45 (CH_2_***C***H_2_CH_3_), 21.33 (OCH_2_***C***H_3_), 27.40 (***C***H_2_CH_2_CH_3_), 42.05, 43.51 (piperazine-C), 60.89 (O***C***H_2_CH_3_), 107.27 (C-5), 148.79, 153.07, 163.99 (C-2, C-6 & C-4), 154.47 (ester C=O). ESI-MS, *m/z* (Rel. Int.): 309.2 (M^−^, 100).

*6-(4-Phenyl-1-piperazinyl)-5-(n-propyl)uracil* (**6f**): ^1^H-NMR (DMSO-d_6_): δ 0.84 (t, 3H, CH_3_, *J* = 7.0 Hz), 1.35-1.37 (m, 2H, C***H***_2_CH_3_), 2.23 (t, 2H, C***H***_2_CH_2_CH_3_, *J* = 7.0 Hz), 3.13 (s, 4H, piperazine-H), 3.25 (s, 4H, piperazine-H), 6.82-6.96 (m, 3H, Ar-H), 7.23 (d, 2H, Ar-H, *J* = 6.5 Hz), 8.28 (br. s, 2H, NH). ^13^C-NMR: 14.20 (CH_3_), 22.02 (***C***H_2_CH_3_), 28.12 (***C***H_2_CH_2_CH_3_), 44.04, 47.34 (piperazine-C), 106.55 (C-5), 116.25, 119.93, 129.49, 151.10 (Ar-C), 153.0, 155.08, 165.01 (C-2, C-6 & C-4). ESI-MS, *m/z* (Rel. Int.): 315.2 (M^+^, 100).

*6-[4-(2-Methoxyphenyl)-1-piperazinyl)]-5-(n-propyl)uracil* (**6g**): ^1^H-NMR (DMSO-d_6_): δ 0.84 (t, 3H, CH_3_, *J* = 7.0 Hz), 1.35-1.40 (m, 2H, C***H***_2_CH_3_), 2.23 (t, 2H, C***H***_2_CH_2_CH_3_, *J* = 7.0 Hz), 3.10 (s, 4H, piperazine-H), 3.15 (s, 4H, piperazine-H), 3.78 (s, 3H, OCH_3_), 6.89-7.0 (m, 4H, Ar-H), 8.35 (br. s, 2H, NH). ^13^C-NMR: 14.27 (CH_3_), 22.09 (***C***H_2_CH_3_), 28.17 (***C***H_2_CH_2_CH_3_), 44.47, 49.03 (piperazine-C), 55.81 (OCH_3_), 106.09 (C-5), 112.37, 118.61, 121.30, 123.46, 141.07, 152.39 (Ar-C), 153.10, 155.93, 165.05 (C-2, C-6 & C-4). ESI-MS, *m/z* (Rel. Int.): 345.2 (M^+^, 100).

*6-[4-(3-Trifluoromethylphenyl)-1-piperazinyl)]-5-(n-propyl)uracil* (**6h**): ^1^H-NMR (DMSO-d_6_): δ 0.84 (t, 3H, CH_3_, *J* = 7.0 Hz), 1.36-1.38 (m, 2H, C***H***_2_CH_3_), 2.24 (t, 2H, C***H***_2_CH_2_CH_3_, *J* = 7.0 Hz), 3.09 (s, 4H, piperazine-H), 3.32 (s, 4H, piperazine-H), 7.09-7.24 (m, 3H, Ar-H), 7.41-7.43 (m, 1H, Ar-H), 7.92 (br. s, 2H, NH). ^13^C-NMR: 14.14 (CH_3_), 21.90 (***C***H_2_CH_3_), 27.99 (***C***H_2_CH_2_CH_3_), 44.25, 47.19 (piperazine-C), 107.16 (C-5), 111.70, 115.50, 119.43, 123.74, 125.93, 130.47, 150.77 (Ar-C & CF_3_), 151.49, 154.14, 164.70 (C-2, C-6 & C-4). ESI-MS, *m/z* (Rel. Int.): 383.2 (M^+^, 100).

*6-(4-Phenyl-1-piperazinyl)-5-isopropyluracil* (**6i**): ^1^H-NMR (DMSO-d_6_): δ 1.16 (d, 6H, CH_3_, *J* = 6.5 Hz), 2.50 (d, 1H, CH, *J* = 6.5 Hz), 3.07 (s, 4H, piperazine-H), 3.21 (s, 4H, piperazine-H), 6.79-6.95 (m, 3H, Ar-H), 7.21-7.22 (m, 2H, Ar-H), 8.06 (br. s, 2H, NH). ^13^C-NMR: 20.58 (CH_3_), 27.75 (CH), 44.52, 47.92 (piperazine-C), 111.26 (C-5), 116.15, 119.76, 129.43, 149.63 (Ar-C), 151.33, 153.80, 164.05 (C-2, C-6 & C-4). ESI-MS, *m/z* (Rel. Int.): 315.2 (M^+^, 100).

*6-[4-(2-Methoxyphenyl)-1-piperazinyl)]-5-isopropyluracil* (**6i**): ^1^H-NMR (DMSO-d_6_): δ 1.17 (d, 6H, CH_3_, *J* = 7.0 Hz), 2.51-2.55 (m, 1H, CH), 2.92-3.09 (m, 8H, piperazine-H), 3.79 (s, 3H, OCH_3_), 6.99-7.42 (m, 4H, Ar-H), 8.11 (br. s. 2H, NH). ^13^C-NMR: 20.18 (***C***H_3_), 27.33 (CH), 44.12, 48.76 (piperazine-C), 55.32 (OCH_3_), 110.06 (C-5), 111.93, 118.11, 120.82, 122.90, 140.70, 150.79 (Ar-C), 151.93, 153.93, 163.82 (C-2, C-6 & C-4). ESI-MS, *m/z* (Rel. Int.): 345.2 (M^+^, 100).

### 3.5. 8-Alkyltetrazolo[1,5-F]pyrimidine-5,7(3H,6H)-dione **7a-c**

A solution of sodium azide (0.7 gm, 0.011 mol) in water (3.0 mL) was added to a solution of the appropriate 5-alkyl-6-chlorouracil **6a**-**c** (0.01 mol) in acetic acid (5 mL), and the mixture was heated under reflux for 6 hours. On cooling, water (10 mL) was added and the mixture was stirred for 30 minutes. The separated crude product was filtered, washed with cold water, dried and crystallized from ethanol.

*8-Ethyltetrazolo[1,5-f]pyrimidine-5,7(3H,6H)-dione* (**7a**): ^1^H-NMR (DMSO-d_6_): δ 0.95 (t, 3H, CH_2_C***H***_3_, *J* = 7.5 Hz), 2.29 (q, 2H, C***H***_2_CH_3_, *J* = 7.5 Hz), 11.32 (s, 1H, NH), 11.82 (s, 1H, NH). ^13^C-NMR: 18.06 (CH_2_***C***H_3_), 19.08 (***C***H_2_CH_3_), 111.86 (C-8), 141.08 (C-9), 150.10 (C-5), 163.20 (C-7). ESI-MS, *m/z* (Rel. Int.): 180.1 (M^−^, 100).

*8-(n-Propyl)tetrazolo[1,5-f]pyrimidine-5,7(3H,6H)-dione* (**7b**): ^1^H-NMR (DMSO-d_6_): δ 0.85 (t, 3H, CH_2_CH_2_C***H***_3_, *J* = 7.5 Hz), 1.37-1.44 (m, 2H, CH_2_C***H***_2_CH_3_), 2.26 (t, 2H, C***H***_2_CH_2_CH_3_, *J* = 7.5 Hz), 11.31 (s, 1H, NH), 11.83 (s, 1H, NH). ^13^C-NMR: 14.05 (CH_2_CH_2_***C***H_3_), 21.45 (CH_2_***C***H_2_CH***_3_***), 27.46 (***C***H_2_CH_2_CH_3_), 110.39 (C-8), 141.49 (C-9), 150.10 (C-5), 163.35 (C-7). ESI-MS, *m/z* (Rel. Int.): 195.1 (M^−^, 100).

*8-Isopropyltetrazolo[1,5-f]pyrimidine-5,7(3H,6H)-dione* (**7c**): ^1^H-NMR (DMSO-d_6_): δ 1.17 (d, 6H, CH_3_, *J* = 7.0 Hz), 2.99-3.03 (m, 1H, CH), 11.23 (s, 1H, NH), 11.75 (s, 1H, NH). ^13^C-NMR: 20.13 (CH_3_), 27.56 (CH), 114.33 (C-8), 140.59 (C-9), 150.0 (C-5), 162.72 (C-7). ESI-MS, *m/z* (Rel. Int.): 195.1 (M^−^, 100).

### 3.6. Determination of the Antimicrobial Activity by the Agar Disc-Diffusion Method [30]

Sterile filter paper discs (8 mm diameter) were moistened with the compound solution in dimethylsulphoxide of specific concentration (200 μg/disc), the antibacterial antibiotics gentamicin and ampicillin trihydrate (100 μg/disc) and the antifungal drug clotrimazole (100 μg/disc) were carefully placed on the agar culture plates that had been previously inoculated separately with the microorganisms. The plates were incubated at 37 °C, and the diameter of the growth inhibition zones were measured after 24 hours in case of bacteria and 48 h in case of *Candida albicans*.

### 3.7. Determination of Minimal Inhibitory Concentration (MIC) [31]

Compound **6h**, gentamicin and ampicillin trihydrate were dissolved in dimethylsulphoxide at a concentration of 128 μg/mL. Twofold serial dilutions of the solution were then prepared (128, 64, 32, …, 0.5 μg/mL). The microorganism suspensions at 106 CFU/mL (colony forming unit/ml) concentrations were inoculated to the corresponding wells. The plates were incubated at 36 °C for 24 and 48 h for the bacteria and *Candida albicans*, respectively. The MIC values were determined as the lowest concentration that completely inhibited visible growth of the microorganism as detected by unaided eye.

## 4. Conclusions

In this study, the synthesis and antimicrobial testing of 5-alkyl-6-(4-substituted-1-piperazinyl)uracils **6a**-**j**, and 8-alkyltetrazolo[1,5-*f*]pyrimidine-5,7(3*H*,6*H*)-diones **7a**-**c**, is described. The compound 6-[4-(3-trifluoromethylphenyl)-1-piperazinyl)]-5-(*n*-propyl)uracil (**6h**) was proven to possess potent broad-spectrum antibacterial activity against certain strains of pathogenic bacteria, although the mechanism of the biological activity needs further investigation, which is in progress.

## Supplementary Materials

Supplementary File 1
