# Supplementary material for: Synthesis and Antimicrobial Activity of Some Novel 5-Alkyl-6-Substituted Uracils and Related Derivatives
Source: Molecules. 2011 Jun 8;16(6):4764–74. doi: 10.3390/molecules16064764 (PMC6264406; doi:10.3390/molecules16064764)
Supplement: Supplementary File 1 [file molecules-16-04764-s001.zip › supplementary/IR.pdf]

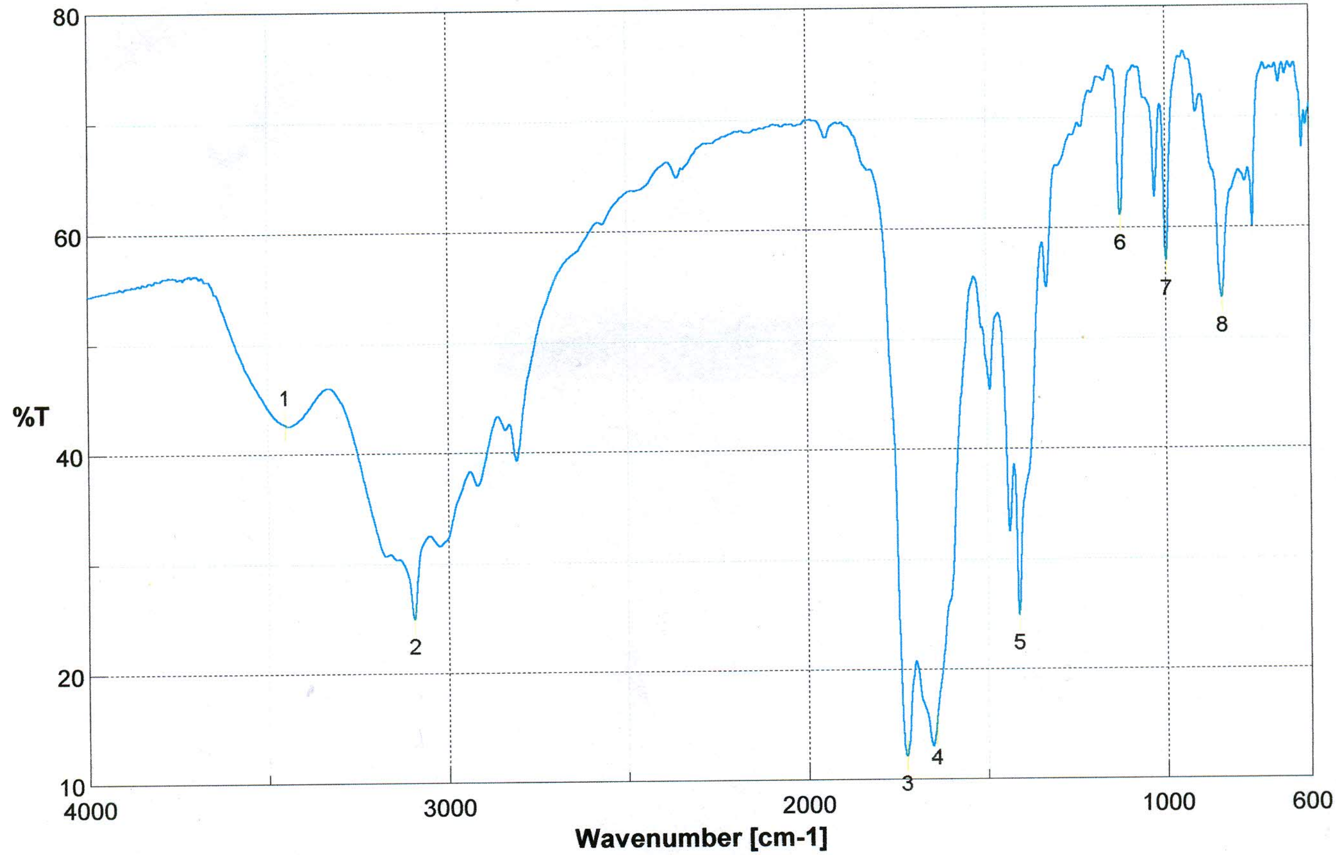

Accumulation	Auto (20)
Resolution	4 cm-1
Zero Filling	ON
Apodization	Cosine
Gain	Auto (2)
Scanning Speed	Auto (2 mm/sec)
Date/Time	3/3/04 9:57AM
Update	3/3/04 10:01AM
Operator	hasson
File Name	Dr. Ali -8.jws
Sample Name	Polystyrene
Comment	tesr air as sample17dec03

No.	cm-1	%T	No.	cm-1	%T	No.	cm-1	%T
1	3451.96	42.6307	2	3095.19	24.932	3	1727.91	12.1387
4	1645.95	14.5281	5	1412.6	24.9005	6	1126.22	61.0959
7	998.946	56.9483	8	843.704	53.5911			

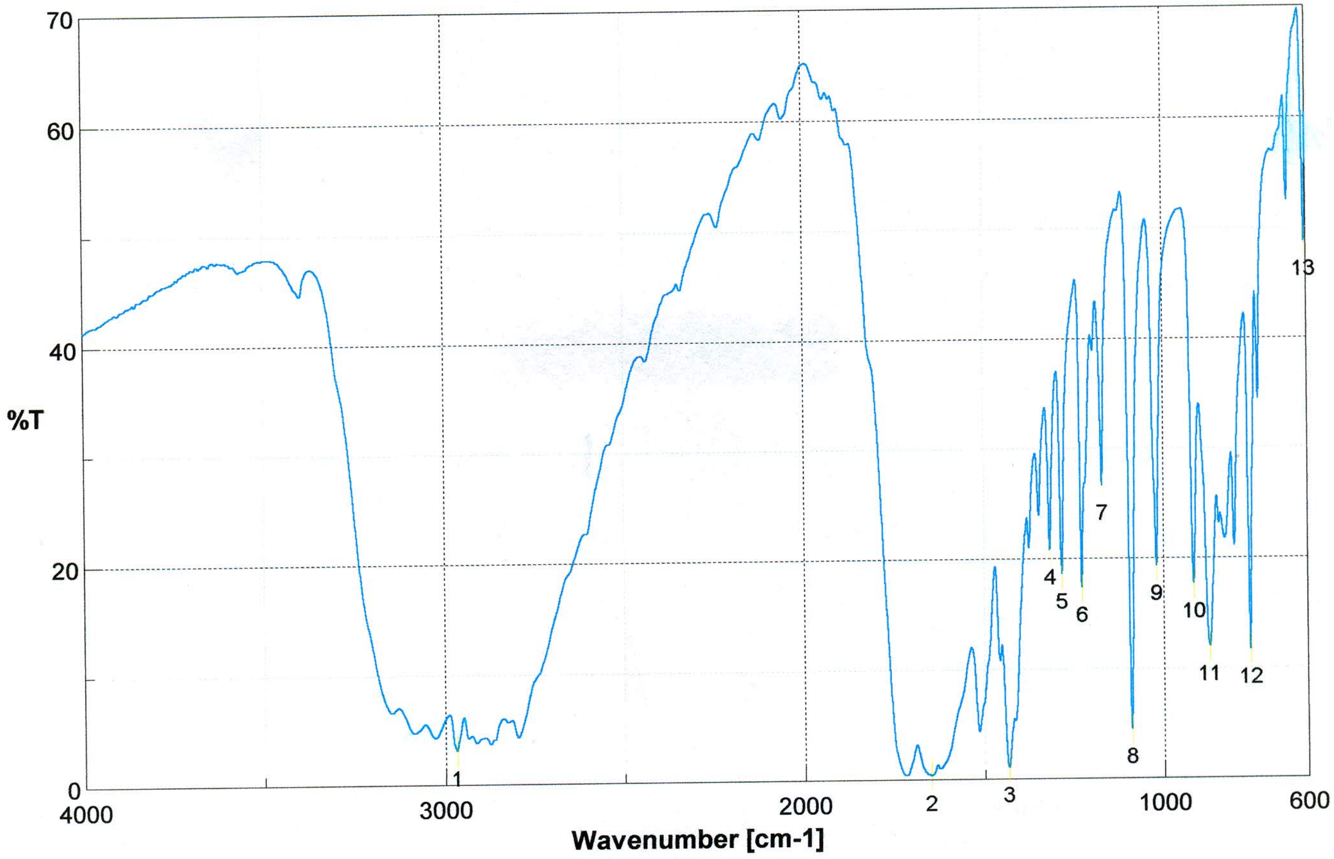

Accumulation	Auto (32)
Resolution	4 cm-1
Zero Filling	ON
Apodization	Cosine
Gain	Auto (2)
Scanning Speed	Auto (2 mm/sec)
Date/Time	3/2/04 3:30PM
Update	3/2/04 3:32PM
Operator	hasson
File Name	Dr. Ali -4.jws
Sample Name	Polystyrene
Comment	tesr air as sample17dec03

No.	cm-1	%T	No.	cm-1	%T	No.	cm-1	%T
1	2967.91	3.15878	2	1650.77	0.41283	3	1433.82	1.11826
4	1317.14	20.8569	5	1283.39	18.6443	6	1228.43	17.4094
7	1171.54	26.6493	8	1090.55	4.5118	9	1020.16	19.3398
10	916.986	17.6609	11	873.596	11.9985	12	759.816	11.7136
13	604.574	48.681						

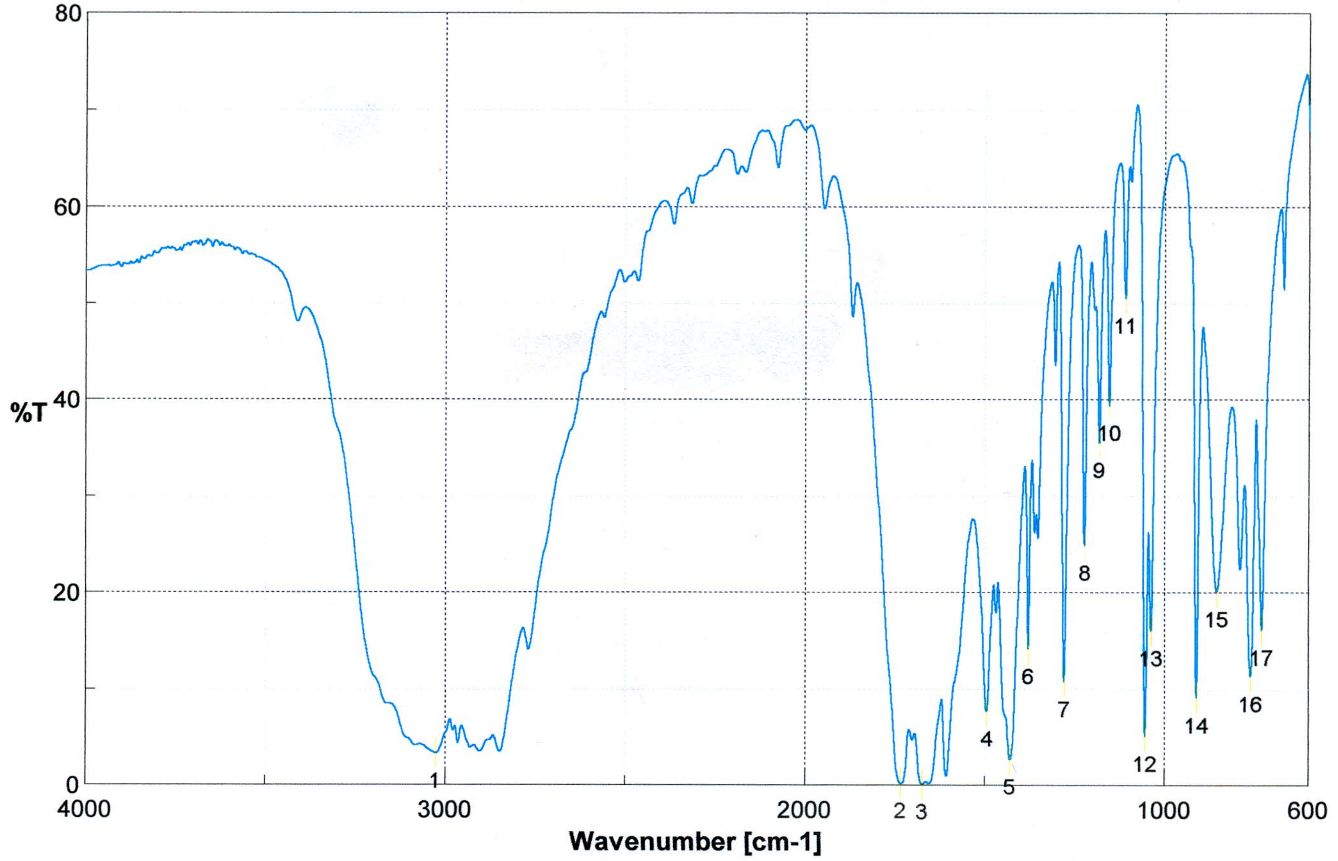

Accumulation	Auto (27)
Resolution	4 cm-1
Zero Filling	ON
Apodization	Cosine
Gain	Auto (2)
Scanning Speed	Auto (2 mm/sec)
Date/Time	3/2/04 3:42PM
Update	3/2/04 3:45PM
Operator	hasson
File Name	Dr. Ali -5.jws
Sample Name	Polystyrene
Comment	tesr air as sample17dec03

No.	cm-1	%T	No.	cm-1	%T	No.	cm-1	%T
1	3026.73	3.34664	2	1733.69	0.233476	3	1672.95	0.119702
4	1495.53	7.71553	5	1429.96	2.72046	6	1379.82	14.1961
7	1280.5	10.7766	8	1223.61	24.861	9	1183.11	35.4988
10	1155.15	39.3594	11	1109.83	50.5252	12	1054.87	5.10382
13	1038.48	15.9996	14	912.165	9.13084	15	856.239	20.1161
16	761.744	11.2871	17	729.925	16.1054			
