# Supplementary material for: Synthesis and Antimicrobial Activity of Some Novel 5-Alkyl-6-Substituted Uracils and Related Derivatives
Source: Molecules. 2011 Jun 8;16(6):4764–74. doi: 10.3390/molecules16064764 (PMC6264406; doi:10.3390/molecules16064764)
Supplement: Supplementary File 1 [file molecules-16-04764-s001.zip › supplementary/molecules-8879-supplementary.pdf]

H1 PY-1/DMSO
MMJABAL

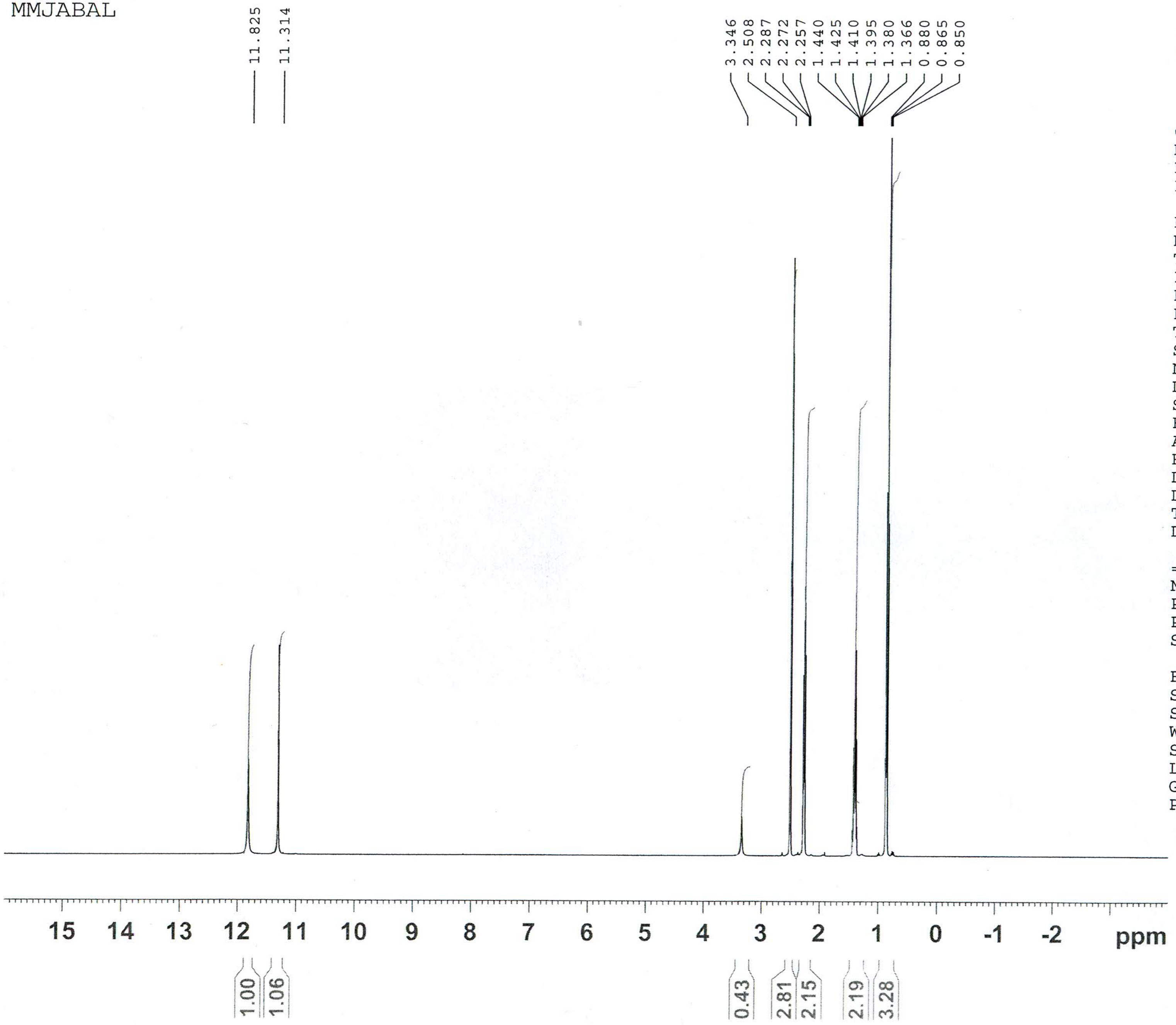

3

BRUKER

H1 PY-1/DMSO
MMJABAL

Current Data Parameters

NAME	drelemam-PY-1
EXPNO	1
PROCNO	1

F2 - Acquisition Parameters

Date_	20040508
Time	9.43
INSTRUM	av500
PROBHD	5 mm BBO BB-1H
PULPROG	zg30
TD	65536
SOLVENT	DMSO
NS	16
DS	0
SWH	10000.000 Hz
FIDRES	0.152588 Hz
AQ	3.2769001 sec
RG	143.7
DW	50.000 usec
DE	6.00 usec
TE	300.0 K
D1	1.00000000 sec

======== CHANNEL f1 ========

NUC1	1H
P1	10.40 usec
PL1	-3.00 dB
SFO1	500.1330008 MHz

F2 - Processing parameters

SI	32768
SF	500.1300000 MHz
WDW	EM
SSB	0
LB	0.30 Hz
GB	0
PC	1.40

H1 PY-1/DMSO
MMJABAL

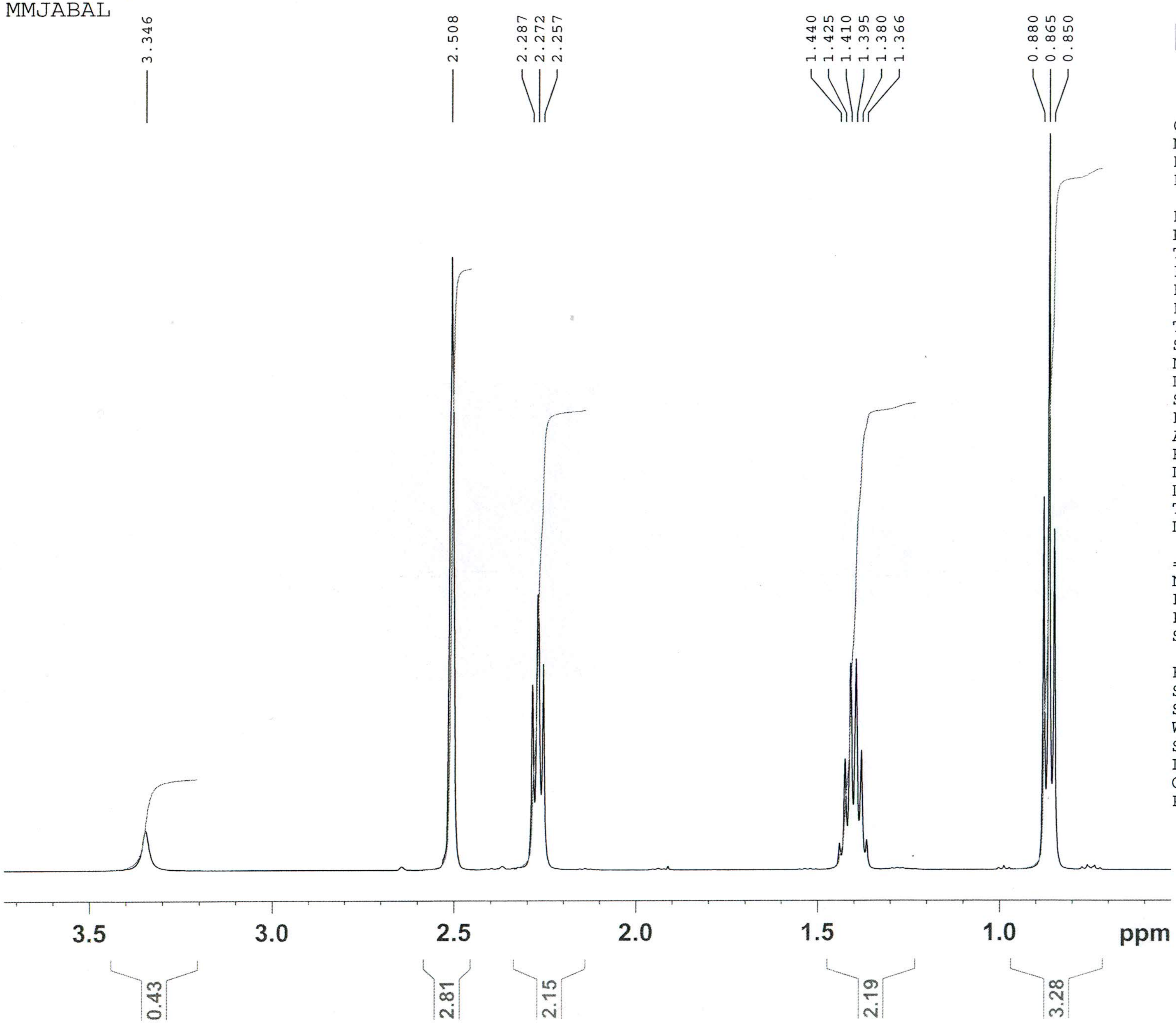

BRUKER

H1 PY-1/DMSO
MMJABAL

```
Current Data Parameters
NAME           drelemam-PY-1
EXPNO                      1
PROCNO                     1

F2 - Acquisition Parameters
Date_               20040508
Time                    9.43
INSTRUM                av500
PROBHD       5 mm BBO BB-1H
PULPROG                 zg30
TD                     65536
SOLVENT                 DMSO
NS                        16
DS                         0
SWH            10000.000 Hz
FIDRES          0.152588 Hz
AQ             3.2769001 sec
RG                     143.7
DW                50.000 usec
DE                  6.00 usec
TE                   300.0 K
D1            1.00000000 sec

======== CHANNEL f1 ========
NUC1                      1H
P1                10.40 usec
PL1                 -3.00 dB
SFO1         500.1330008 MHz

F2 - Processing parameters
SI                     32768
SF           500.1300000 MHz
WDW                       EM
SSB                        0
LB                   0.30 Hz
GB                         0
PC                      1.40
```

C13 PY-1/DMSO
MMJABAL

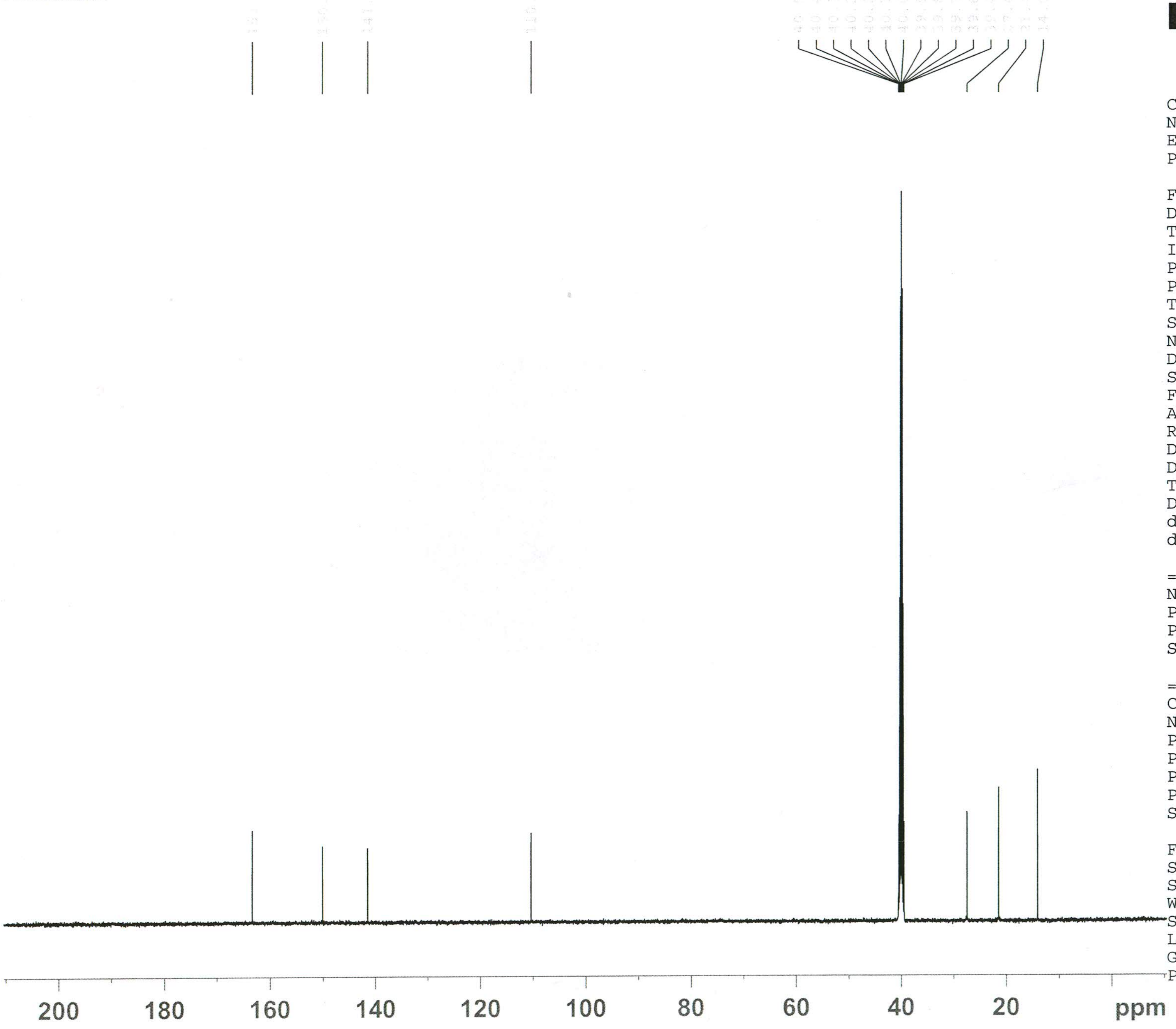

BRUKER

Current Data Parameters

NAME	drelemam-PY-1
EXPNO	2
PROCNO	1

F2 - Acquisition Parameters

Date_	20040508
Time	10.02
INSTRUM	av500
PROBHD	5 mm BBO BB-1H
PULPROG	zgpg30
TD	65536
SOLVENT	DMSO
NS	353
DS	4
SWH	27777.777 Hz
FIDRES	0.423855 Hz
AQ	1.1797160 sec
RG	181
DW	18.000 usec
DE	6.00 usec
TE	300.0 K
D1	2.00000000 sec
d11	0.03000000 sec
d12	0.00002000 sec

======== CHANNEL f1 ========

NUC1	13C
P1	5.80 usec
PL1	-2.00 dB
SFO1	125.7703643 MHz

======== CHANNEL f2 ========

CPDPRG2	waltz16
NUC2	1H
PCPD2	80.00 usec
PL2	-3.00 dB
PL12	15.00 dB
PL13	15.00 dB
SFO2	500.1320005 MHz

F2 - Processing parameters

SI	32768
SF	125.7577890 MHz
WDW	EM
SSB	0
LB	1.00 Hz
GB	0
PC	1.00
