# Supplementary material for: Synthesis and Antimicrobial Activity of Some Novel 5-Alkyl-6-Substituted Uracils and Related Derivatives
Source: Molecules. 2011 Jun 8;16(6):4764–74. doi: 10.3390/molecules16064764 (PMC6264406; doi:10.3390/molecules16064764)
Supplement: Supplementary File 1 [file molecules-16-04764-s001.zip › supplementary/NMR-5b.pdf]

H1 GH-28/DMSO
MMJABAL

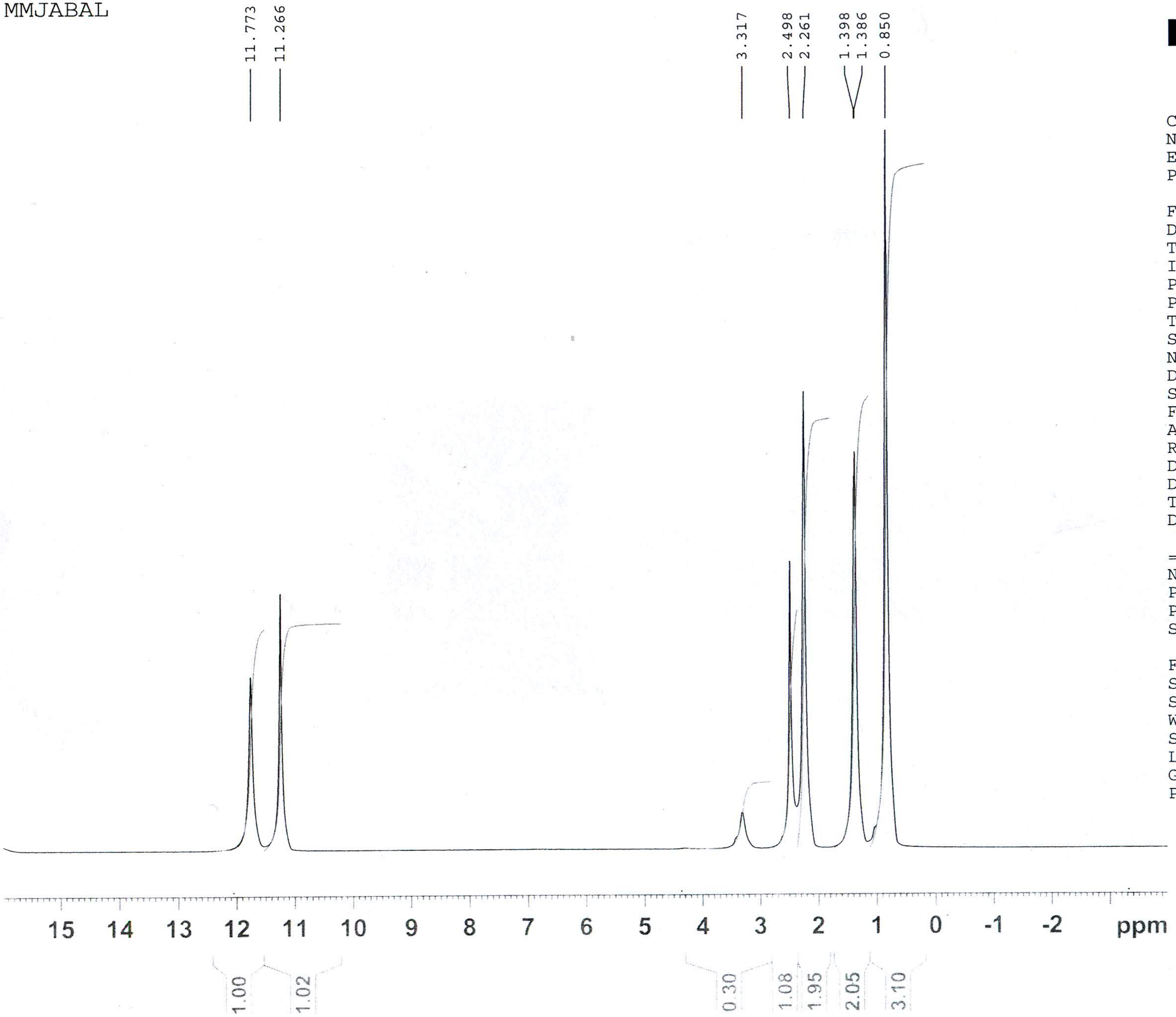

AAT-35

H1 GH-28/DMSO
MMJABAL

Current Data Parameters

NAME	dremam-GH-28
EXPNO	1
PROCNO	1

F2 - Acquisition Parameters

Date_	20090521
Time	13.21
INSTRUM	av500
PROBHD	5 mm BBO BB-1H
PULPROG	zg30
TD	65536
SOLVENT	DMSO
NS	16
DS	0
SWH	10000.000 Hz
FIDRES	0.152588 Hz
AQ	3.2769001 sec
RG	101.6
DW	50.000 usec
DE	6.00 usec
TE	300.0 K
D1	1.00000000 sec

======== CHANNEL f1 ========

NUC1	1H
P1	10.40 usec
PL1	-3.00 dB
SFO1	500.1330008 MHz

F2 - Processing parameters

SI	32768
SF	500.1300000 MHz
WDW	EM
SSB	0
LB	0.30 Hz
GB	0
PC	1.00

(4)

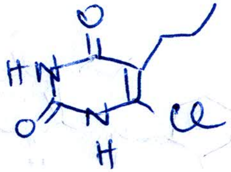

H1 GH-28/DMSO
MMJABAL

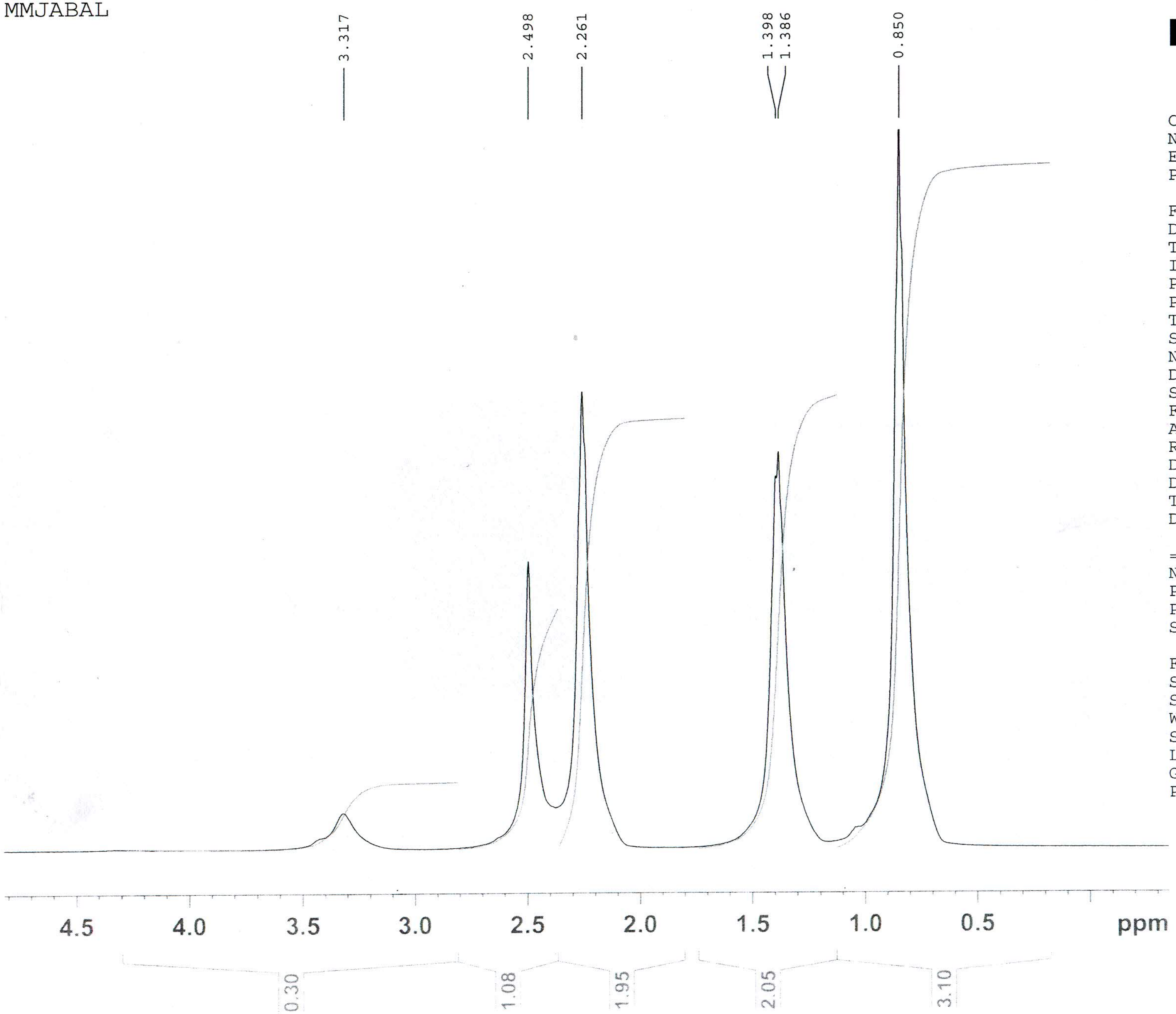

Current Data Parameters
NAME dremam-GH-28
EXPNO 1
PROCNO 1

H1 GH-28/DMSO
MMJABAL

F2 - Acquisition Parameters

Date_	20090521
Time	13.21
INSTRUM	av500
PROBHD	5 mm BBO BB-1H
PULPROG	zg30
TD	65536
SOLVENT	DMSO
NS	16
DS	0
SWH	10000.000 Hz
FIDRES	0.152588 Hz
AQ	3.2769001 sec
RG	101.6
DW	50.000 usec
DE	6.00 usec
TE	300.0 K
D1	1.00000000 sec

======== CHANNEL f1 ========

NUC1	1H
P1	10.40 usec
PL1	-3.00 dB
SFO1	500.1330008 MHz

F2 - Processing parameters

SI	32768
SF	500.1300000 MHz
WDW	EM
SSB	0
LB	0.30 Hz
GB	0
PC	1.00

C13 GH-28/DMSO
MMJABAL

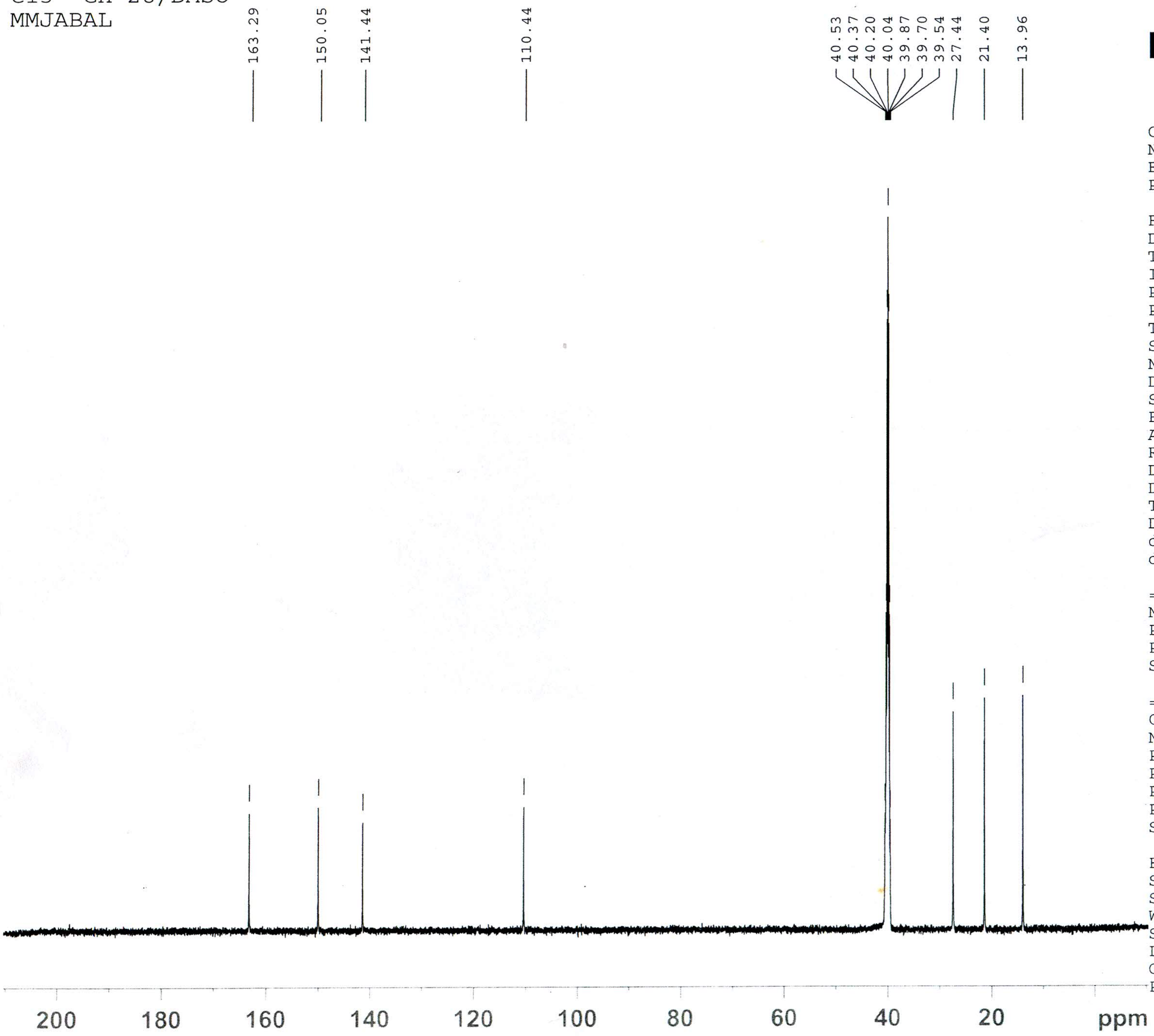

Current Data Parameters

NAME	dremam-GH-28
EXPNO	2
PROCNO	1

F2 - Acquisition Parameters

Date_	20090521
Time	13.31
INSTRUM	av500
PROBHD	5 mm BBO BB-1H
PULPROG	zgpg30
TD	65536
SOLVENT	CDCl3
NS	849
DS	4
SWH	27777.777 Hz
FIDRES	0.423855 Hz
AQ	1.1797160 sec
RG	80.6
DW	18.000 usec
DE	6.00 usec
TE	300.0 K
D1	2.00000000 sec
d11	0.03000000 sec
d12	0.00002000 sec

======== CHANNEL f1 ========

NUC1	13C
P1	5.80 usec
PL1	-2.00 dB
SFO1	125.7703643 MHz

======== CHANNEL f2 ========

CPDPRG2	waltz16
NUC2	1H
PCPD2	80.00 usec
PL2	-3.00 dB
PL12	15.00 dB
PL13	15.00 dB
SFO2	500.1320005 MHz

F2 - Processing parameters

SI	32768
SF	125.7577890 MHz
WDW	EM
SSB	0
LB	1.00 Hz
GB	0
PC	1.00
