# Supplementary material for: Synthesis and Antimicrobial Activity of Some Novel 5-Alkyl-6-Substituted Uracils and Related Derivatives
Source: Molecules. 2011 Jun 8;16(6):4764–74. doi: 10.3390/molecules16064764 (PMC6264406; doi:10.3390/molecules16064764)
Supplement: Supplementary File 1 [file molecules-16-04764-s001.zip › supplementary/NMR-6h.pdf]

H1 GH-34/DMSO
MMJABAL

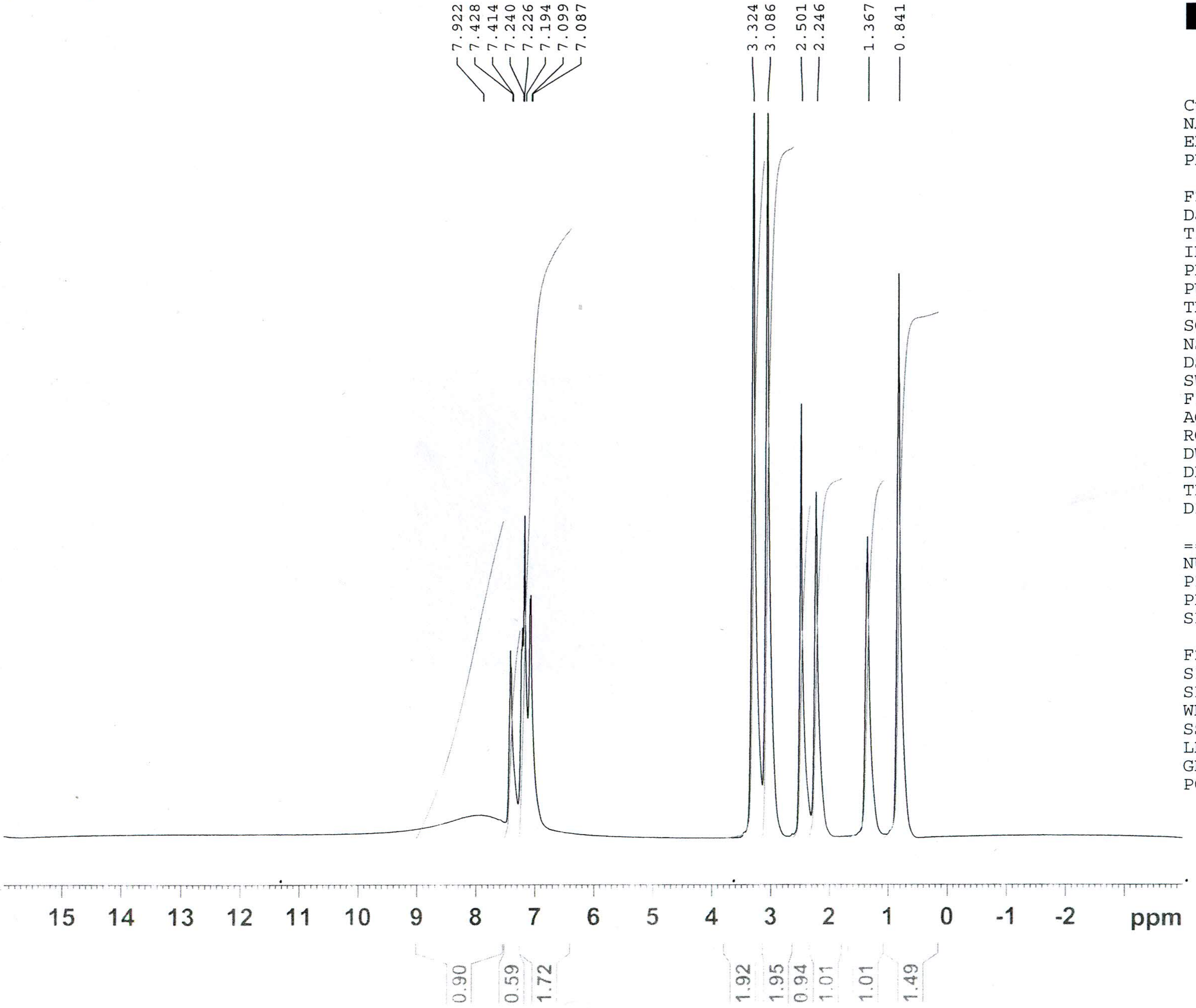

2

BRUKER

H1 GH-34/DMSO
MMJABAL

Current Data Parameters
NAME dremam-GH-34
EXPNO 1
PROCNO 1

F2 - Acquisition Parameters
Date_ 20090616
Time 10.52
INSTRUM av500
PROBHD 5 mm BBO BB-1H
PULPROG zg30
TD 65536
SOLVENT DMSO
NS 16
DS 0
SWH 10000.000 Hz
FIDRES 0.152588 Hz
AQ 3.2769001 sec
RG 90.5
DW 50.000 usec
DE 6.00 usec
TE 300.0 K
D1 1.00000000 sec

======== CHANNEL f1 ========
NUC1 1H
P1 10.40 usec
PL1 -3.00 dB
SFO1 500.1330008 MHz

F2 - Processing parameters
SI 32768
SF 500.1300000 MHz
WDW EM
SSB 0
LB 0.30 Hz
GB 0
PC 1.00

H1 GH-34/DMSO
MMJABAL

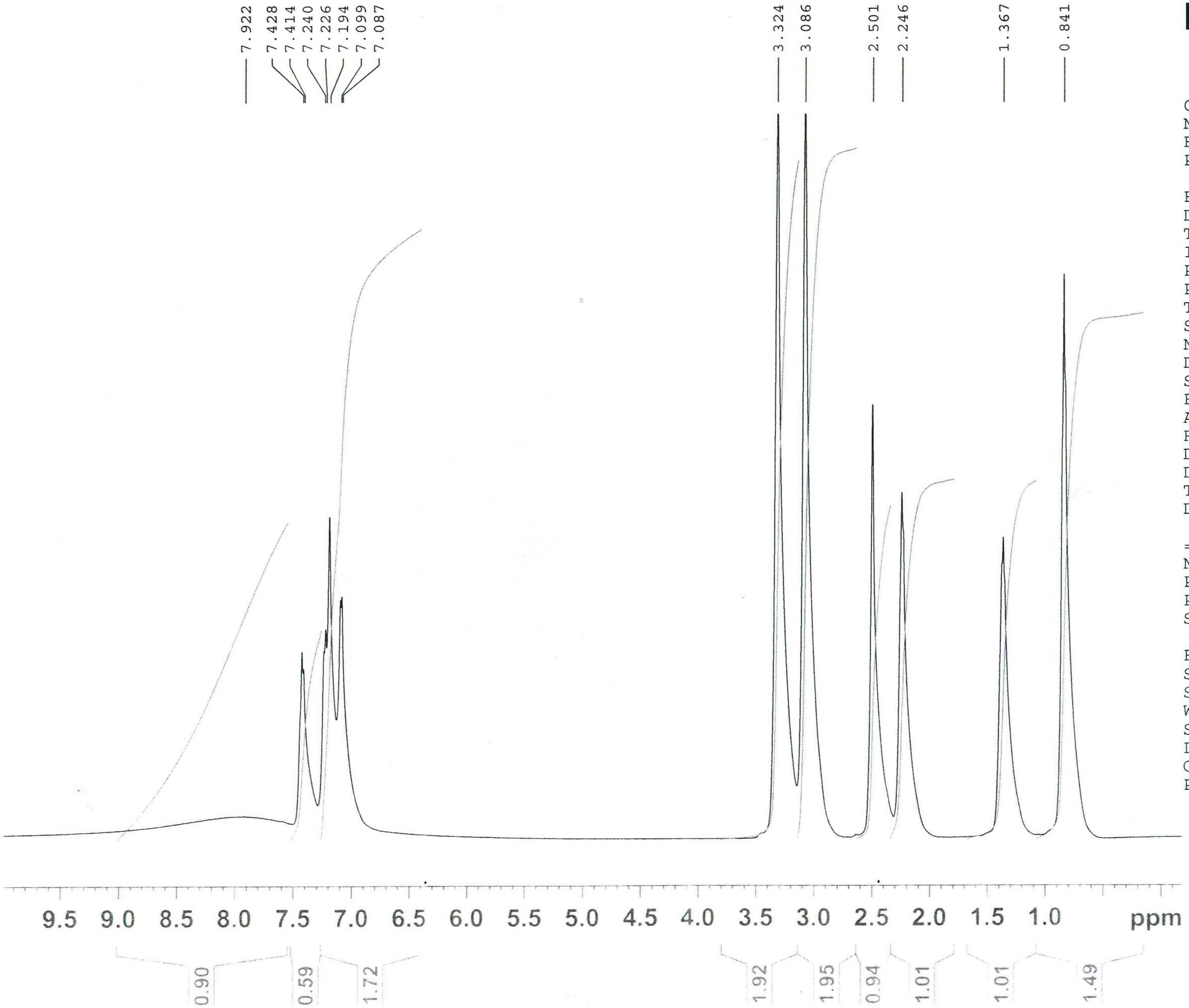

H1 GH-34/DMSO
MMJABAL

Current Data Parameters	
NAME	dremam-GH-34
EXPNO	1
PROCNO	1

F2 - Acquisition Parameters	
Date_	20090616
Time	10.52
INSTRUM	av500
PROBHD	5 mm BBO BB-1H
PULPROG	zg30
TD	65536
SOLVENT	DMSO
NS	16
DS	0
SWH	10000.000 Hz
FIDRES	0.152588 Hz
AQ	3.2769001 sec
RG	90.5
DW	50.000 usec
DE	6.00 usec
TE	300.0 K
D1	1.00000000 sec

======== CHANNEL f1 ========	
NUC1	1H
P1	10.40 usec
PL1	-3.00 dB
SFO1	500.1330008 MHz

F2 - Processing parameters	
SI	32768
SF	500.1300000 MHz
WDW	EM
SSB	0
LB	0.30 Hz
GB	0
PC	1.00

C13 GH-34/DMSO
MMJABAL

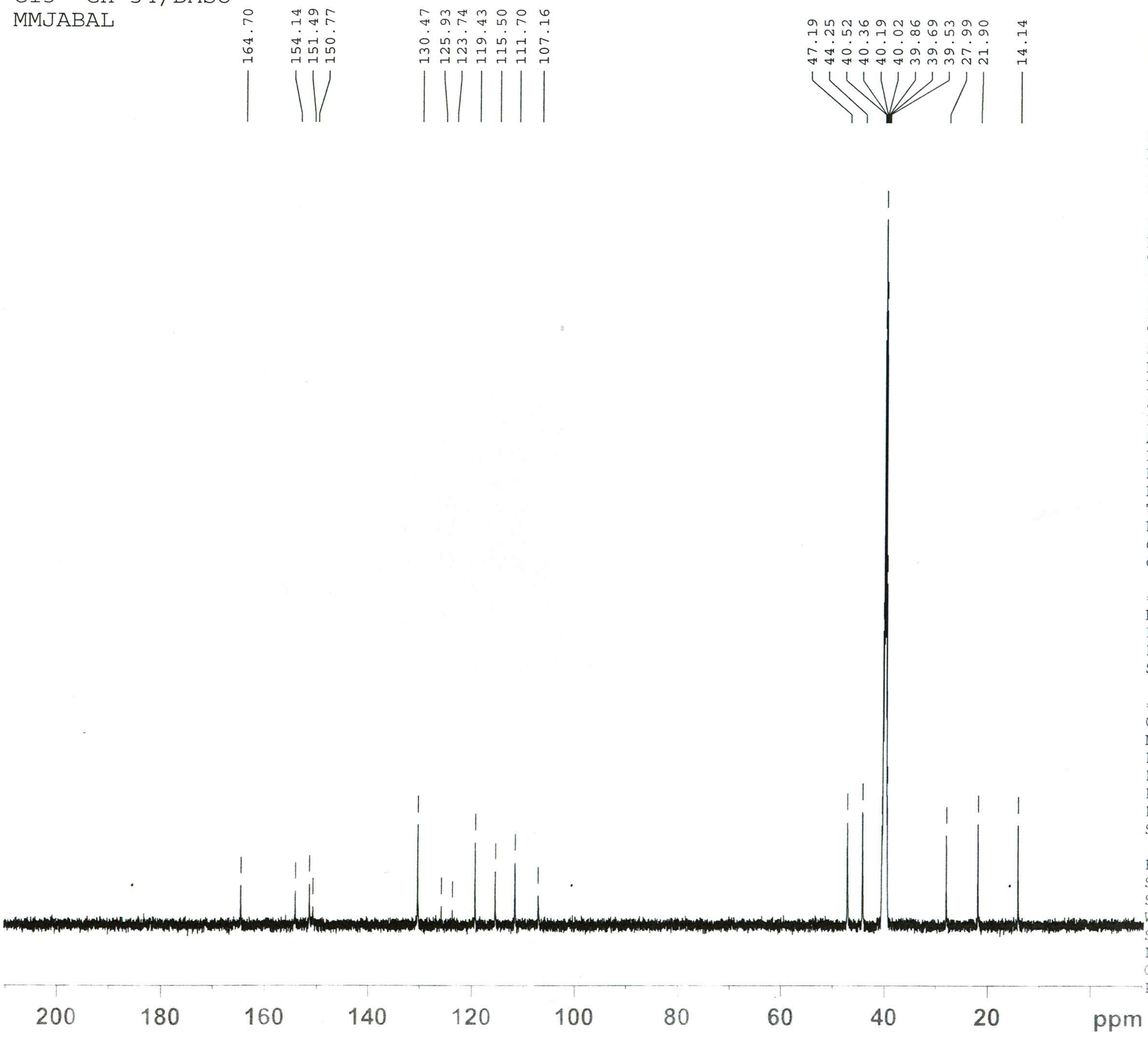

Current Data Parameters
NAME dremam-GH-34
EXPNO 2
PROCNO 1

F2 - Acquisition Parameters
Date_ 20090616
Time 10.56
INSTRUM av500
PROBHD 5 mm BBO BB-1H
PULPROG zgpg30
TD 65536
SOLVENT DMSO
NS 295
DS 4
SWH 27777.777 Hz
FIDRES 0.423855 Hz
AQ 1.1797160 sec
RG 203.2
DW 18.000 usec
DE 6.00 usec
TE 300.0 K
D1 2.00000000 sec
d11 0.03000000 sec
d12 0.00002000 sec

======== CHANNEL f1 ========
NUC1 13C
P1 5.80 usec
PL1 -2.00 dB
SFO1 125.7703643 MHz

======== CHANNEL f2 ========
CPDPRG2 waltz16
NUC2 1H
PCPD2 80.00 usec
PL2 -3.00 dB
PL12 15.00 dB
PL13 15.00 dB
SFO2 500.1320005 MHz

F2 - Processing parameters
SI 32768
SF 125.7577890 MHz
WDW EM
SSB 0
LB 1.00 Hz
GB 0
PC 1.40

C13 GH-34/DMSO
MMJABAL

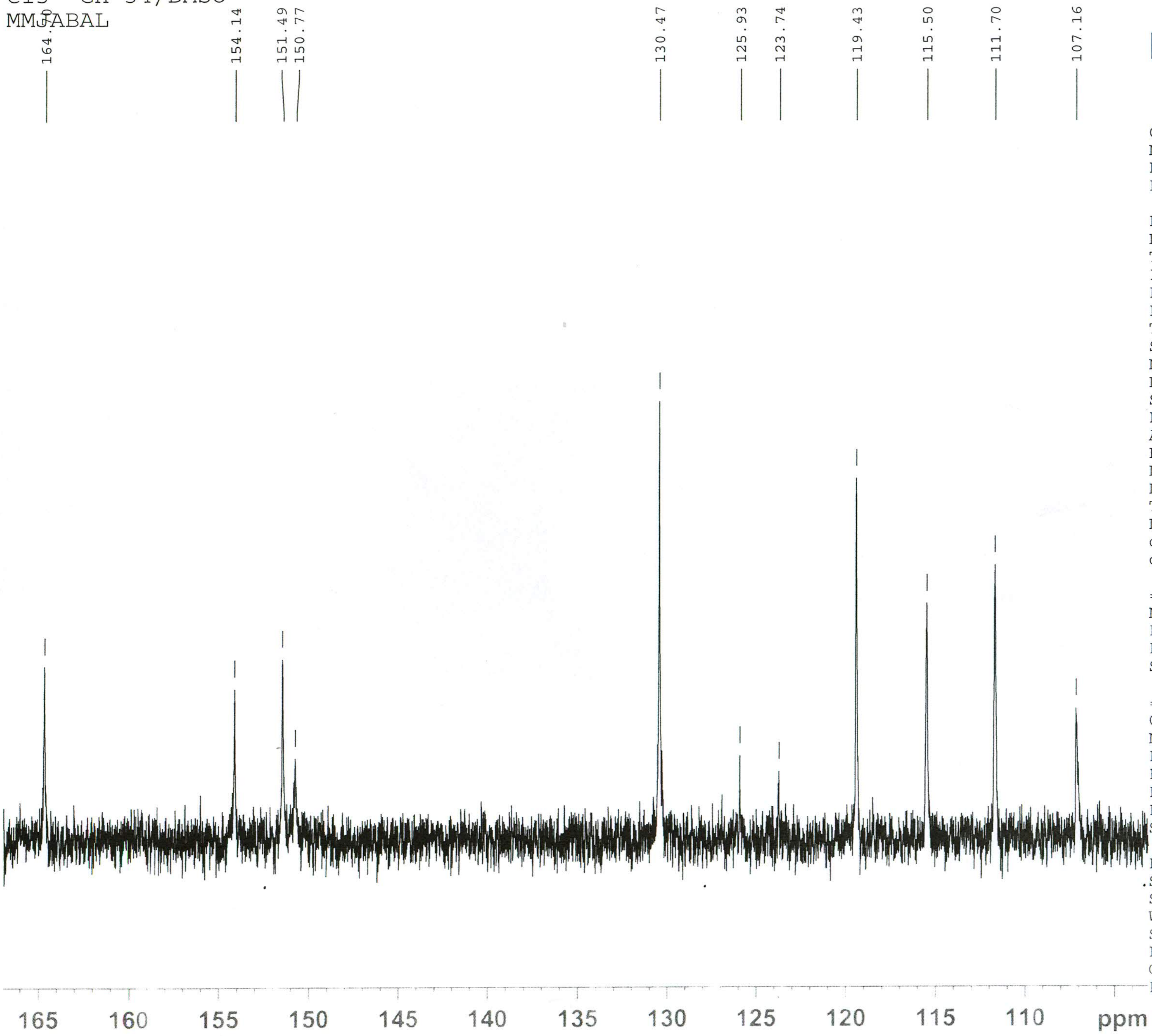

BRUKER

Current Data Parameters
NAME dremam-GH-34
EXPNO 2
PROCNO 1

F2 - Acquisition Parameters
Date_ 20090616
Time 10.56
INSTRUM av500
PROBHD 5 mm BBO BB-1H
PULPROG zgpg30
TD 65536
SOLVENT DMSO
NS 295
DS 4
SWH 27777.777 Hz
FIDRES 0.423855 Hz
AQ 1.1797160 sec
RG 203.2
DW 18.000 usec
DE 6.00 usec
TE 300.0 K
D1 2.00000000 sec
d11 0.03000000 sec
d12 0.00002000 sec

======== CHANNEL f1 ========
NUC1 13C
P1 5.80 usec
PL1 -2.00 dB
SFO1 125.7703643 MHz

======== CHANNEL f2 ========
CPDPRG2 waltz16
NUC2 1H
PCPD2 80.00 usec
PL2 -3.00 dB
PL12 15.00 dB
PL13 15.00 dB
SFO2 500.1320005 MHz

F2 - Processing parameters
SI 32768
SF 125.7577890 MHz
WDW EM
SSB 0
LB 1.00 Hz
GB 0
PC 1.40

C13 GH-34/DMSO
MMJABAL

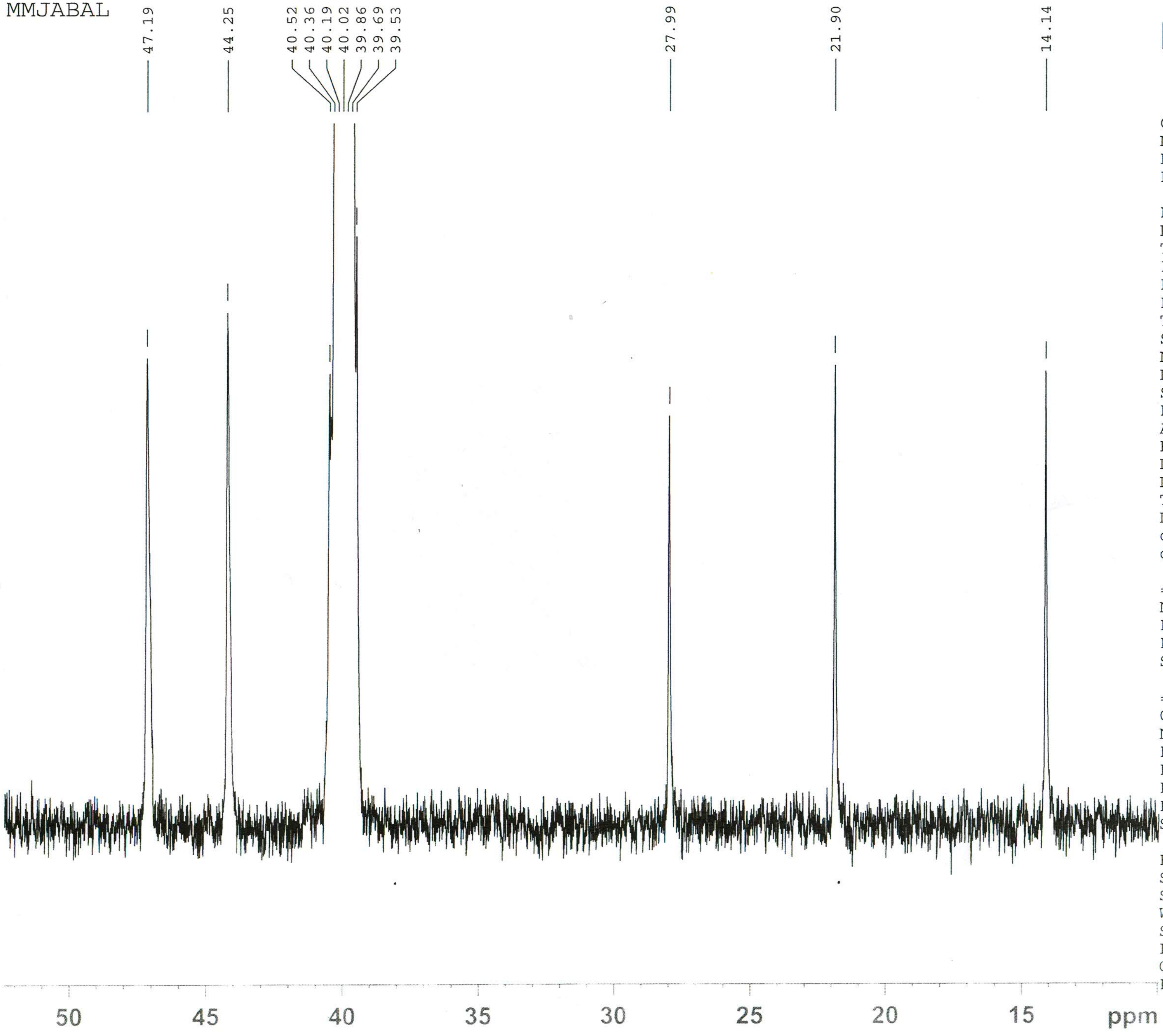

BRUKER

Current Data Parameters
NAME dremam-GH-34
EXPNO 2
PROCNO 1

F2 - Acquisition Parameters
Date_ 20090616
Time 10.56
INSTRUM av500
PROBHD 5 mm BBO BB-1H
PULPROG zgpg30
TD 65536
SOLVENT DMSO
NS 295
DS 4
SWH 27777.777 Hz
FIDRES 0.423855 Hz
AQ 1.1797160 sec
RG 203.2
DW 18.000 usec
DE 6.00 usec
TE 300.0 K
D1 2.00000000 sec
d11 0.03000000 sec
d12 0.00002000 sec

======== CHANNEL f1 ========
NUC1 13C
P1 5.80 usec
PL1 -2.00 dB
SFO1 125.7703643 MHz

======== CHANNEL f2 ========
CPDPRG2 waltz16
NUC2 1H
PCPD2 80.00 usec
PL2 -3.00 dB
PL12 15.00 dB
PL13 15.00 dB
SFO2 500.1320005 MHz

F2 - Processing parameters
SI 32768
SF 125.7577890 MHz
WDW EM
SSB 0
LB 1.00 Hz
GB 0
PC 1.40
