# Supplementary material for: Synthesis and Antimicrobial Activity of Some Novel 5-Alkyl-6-Substituted Uracils and Related Derivatives
Source: Molecules. 2011 Jun 8;16(6):4764–74. doi: 10.3390/molecules16064764 (PMC6264406; doi:10.3390/molecules16064764)
Supplement: Supplementary File 1 [file molecules-16-04764-s001.zip › supplementary/NMR-7a.pdf]

H1 PY-3/DMSO
MMJABAL

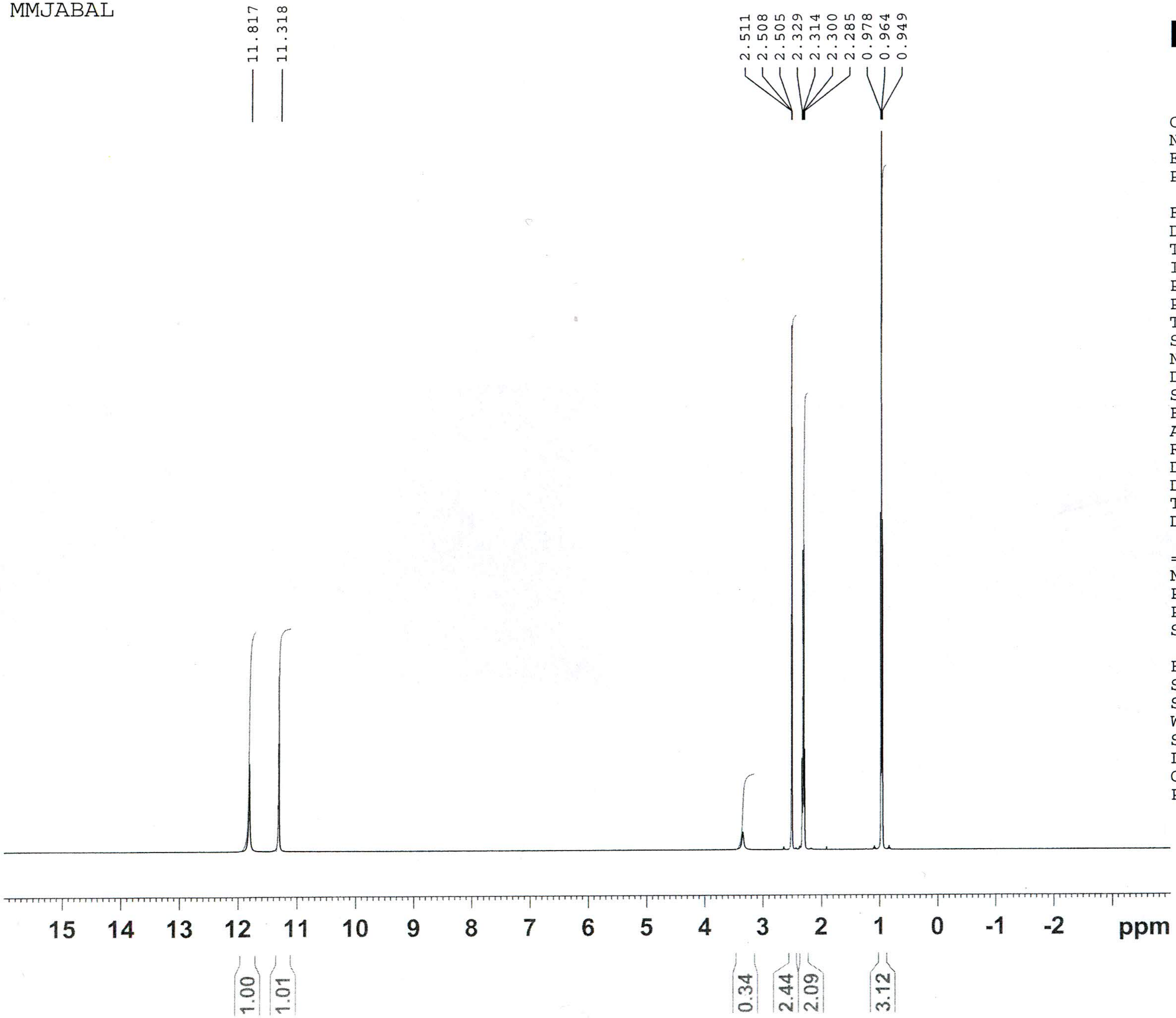

H1 PY-3/DMSO
MMJABAL

5

Current Data Parameters
NAME drelemam-PY-3
EXPNO 1
PROCNO 1

F2 - Acquisition Parameters

Date_	20040512
Time	9.08
INSTRUM	av500
PROBHD	5 mm BBO BB-1H
PULPROG	zg30
TD	65536
SOLVENT	DMSO
NS	16
DS	0
SWH	10000.000 Hz
FIDRES	0.152588 Hz
AQ	3.2769001 sec
RG	161.3
DW	50.000 usec
DE	6.00 usec
TE	300.0 K
D1	1.00000000 sec

======== CHANNEL f1 ========

NUC1	1H
P1	10.40 usec
PL1	-3.00 dB
SFO1	500.1330008 MHz

F2 - Processing parameters

SI	32768
SF	500.1300000 MHz
WDW	EM
SSB	0
LB	0.30 Hz
GB	0
PC	1.40

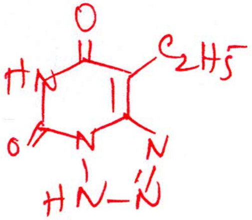

H1 PY-3/DMSO
MMJABAL

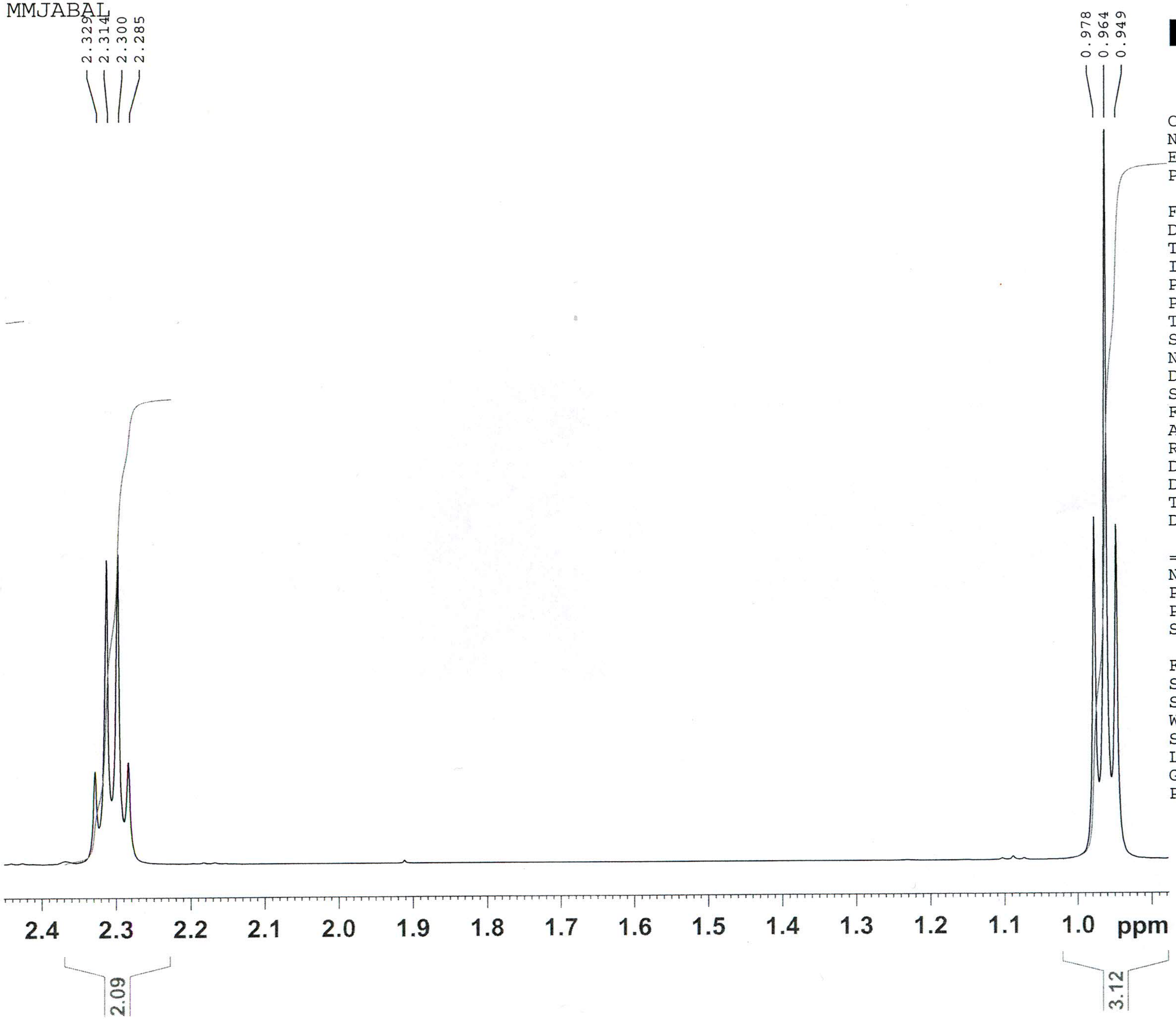

H1 PY-3/DMSO
MMJABAL

Current Data Parameters
NAME drelemam-PY-3
EXPNO 1
PROCNO 1

F2 - Acquisition Parameters
Date_ 20040512
Time 9.08
INSTRUM av500
PROBHD 5 mm BBO BB-1H
PULPROG zg30
TD 65536
SOLVENT DMSO
NS 16
DS 0
SWH 10000.000 Hz
FIDRES 0.152588 Hz
AQ 3.2769001 sec
RG 161.3
DW 50.000 usec
DE 6.00 usec
TE 300.0 K
D1 1.00000000 sec

======== CHANNEL f1 ========
NUC1 1H
P1 10.40 usec
PL1 -3.00 dB
SFO1 500.1330008 MHz

F2 - Processing parameters
SI 32768
SF 500.1300000 MHz
WDW EM
SSB 0
LB 0.30 Hz
GB 0
PC 1.40

C13 PY-3/DMSO
MMJABAL

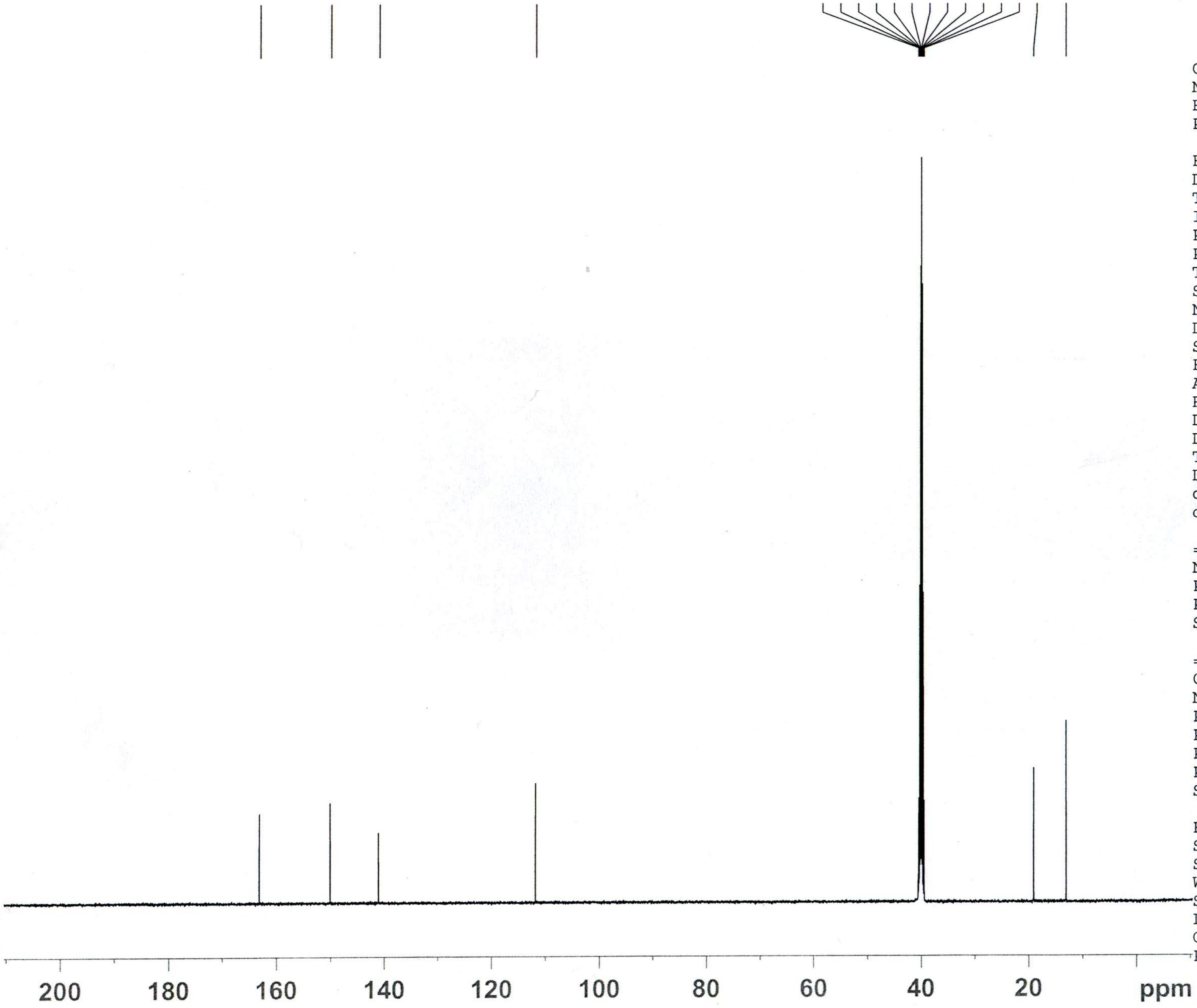

Current Data Parameters

NAME	drelemam-PY-3
EXPNO	2
PROCNO	1

F2 - Acquisition Parameters

Date_	20040512
Time	9.43
INSTRUM	av500
PROBHD	5 mm BBO BB-1H
PULPROG	zgpg30
TD	65536
SOLVENT	DMSO
NS	636
DS	4
SWH	27777.777 Hz
FIDRES	0.423855 Hz
AQ	1.1797160 sec
RG	812.7
DW	18.000 usec
DE	6.00 usec
TE	300.0 K
D1	2.00000000 sec
d11	0.03000000 sec
d12	0.00002000 sec

======== CHANNEL f1 ========

NUC1	13C
P1	5.80 usec
PL1	-2.00 dB
SFO1	125.7703643 MHz

======== CHANNEL f2 ========

CPDPRG2	waltz16
NUC2	1H
PCPD2	80.00 usec
PL2	-3.00 dB
PL12	15.00 dB
PL13	15.00 dB
SFO2	500.1320005 MHz

F2 - Processing parameters

SI	32768
SF	125.7577890 MHz
WDW	EM
SSB	0
LB	1.00 Hz
GB	0
PC	1.00
